# The effect of age and sex on the expression of GABA signaling components in the human hippocampus and entorhinal cortex

**DOI:** 10.1038/s41598-021-00792-8

**Published:** 2021-11-02

**Authors:** Jayarjun Ethiraj, Thulani Hansika Palpagama, Clinton Turner, Bert van der Werf, Henry John Waldvogel, Richard Lewis Maxwell Faull, Andrea Kwakowsky

**Affiliations:** 1 Centre for Brain Research, Department of Anatomy and Medical Imaging, Faculty of Medical and Health Sciences, School of Medical Sciences, University of Auckland, Auckland, New Zealand; 2 Department of Anatomical Pathology, LabPlus, Auckland City Hospital, Auckland, New Zealand; 3 Department of Epidemiology and Biostatistics, Faculty of Medical and Health Sciences, School of Population Health, University of Auckland, Auckland, New Zealand

**Keywords:** Neural ageing, Synaptic transmission, Neuroscience

## Abstract

Gamma-aminobutyric acid (GABA) is the primary inhibitory neurotransmitter in the nervous system. The GABA signaling system in the brain is comprised of GABA synthesizing enzymes, transporters, GABAA and GABAB receptors (GABA_A_R and GABA_B_R). Alterations in the expression of these signaling components have been observed in several brain regions throughout aging and between sexes in various animal models. The hippocampus is the memory centre of the brain and is impaired in several age-related disorders. It is composed of two main regions: the Cornu Ammonis (CA1-4) and the Dentate Gyrus (DG), which are interconnected with the Entorhinal Cortex (ECx). The age- and sex-specific changes of GABA signaling components in these regions of the human brain have not been examined. This study is the first to determine the effect of age and sex on the expression of GABA signaling components-GABA_A_R α1,2,3,5, β1-3, γ2, GABA_B_R R1 and R2 subunits and the GABA synthesizing enzymes GAD 65/67-in the ECx, and the CA1 and DG regions of the human hippocampus using Western blotting. No significant differences were found in GABA_A_R α1,2,3,5, β1-3, γ2, GABA_B_R R1 and R2 subunit and GAD65/76 expression levels in the ECx, CA1 and DG regions between the younger and older age groups for both sexes. However, we observed a significant negative correlation between age and GABA_A_R α1subunit level in the CA1 region for females; significant negative correlation between age and GABA_A_R β1, β3 and γ2 subunit expression in the DG region for males. In females a significant positive correlation was found between age and GABA_A_R γ2 subunit expression in the ECx and GABA_B_R R2 subunit expression in the CA1 region. The results indicate that age and sex do not affect the expression of GAD 65/67. In conclusion, our results show age- and sex-related GABA_A/B_R subunit alterations in the ECx and hippocampus that might significantly influence GABAergic neurotransmission and underlie disease susceptibility and progression.

## Introduction

Gamma-aminobutyric acid (GABA) is the chief inhibitory neurotransmitter in the mammalian brain. GABA is synthesised from glutamate via the glutamic acid decarboxylase enzyme (GAD) and packaged into synaptic vesicles by the vesicular GABA transporter (VGAT)^1,2^. Once depolarization of the membrane occurs, GABA is released from the vesicles into the synaptic space, where it binds to ionotropic GABA_A_ receptors (GABA_A_Rs) or metabotropic GABA_B_ receptors (GABA_B_Rs)^3,4^. Excess GABA is cleared from the synaptic space by membrane GABA transporters (GATs)^5^. The inhibitory action of GABA is important to maintain the excitatory-inhibitory balance within the brain, which is vital to normal brain function. The expression and function of the GABAergic signaling components are key to optimal GABAergic inhibition in the brain. Previous studies have reported fluctuations in expression levels of GAD, GABA_A/B_R subunits, and GATs with increasing age and between sexes, but this limited knowledge is highly based on animal models that produce inconsistent findings. Recent evidence indicates that the expression of specific GABAergic signaling components displays differences between sexes and is also affected by aging in specific brain regions of the human brain^6,7^. However, the impact of age and sex on GABA signaling have not been studied in the human hippocampus, the memory centre of the brain, which is affected by aging and in age-related disorders^8–10^.

GABA_A_Rs are pentameric structures assembled by five subunits to create a ligand-gated Cl^-^ ion channel pore. Studies have successfully identified and cloned > 20 subunits thus far, including the alpha (α1-6), beta (β1-3), gamma (γ1-3), rho (r1-3), delta, epsilon and theta classes of GABA_A_R subunits^4^. Given the number of subunits present, there are many GABA_A_R subunit combinations that can be formed in theory. GABA_B_Rs are seven-transmembrane-domain G-protein coupled receptors made up of two receptor subunits; GABA_B_R R1 and GABA_B_ R2. The former binds GABA, while the latter is associated with G-protein interaction^3^.

We showed a significant age-related decrease in GABA_A_R α3 subunit expression in the human superior temporal gyrus (STG) of males, however there were no changes in other cortical areas such as the primary sensory and motor cortices, medial temporal gyrus (MTG), inferior temporal gyrus (ITG) and cerebellum^6^. In the hippocampus specifically, there is conflicting evidence, with a recent paper showing no significant changes in levels of GABA_A_R subunits in the mouse^11^, however previous studies have shown an age-related increase in the expression of α1 and γ2 subunits in the rat hippocampus^12–14^. The age-related changes in GABA_A_R subunit expression has been implicated in contributing to ageing-related learning impairments^15–18^.

Age-related decrease in GABA_B_R subunit expression in the inferior colliculus, hippocampus and prefrontal cortex (PFC) was reported in rats^19,20^. Levels of hippocampal GABA_B_ R1 isoforms were similar between the young and old age groups but aged-impaired rats (impaired relative to young) expressed less of both isoforms (GABA_B_ R1a and GABA_B_ R1b) than young. Levels of GABA_B_ R2 were not changed by age or cognitive group (unimpaired or impaired relative to young) in the hippocampus. In contrast, GABABR1 isoforms were similarly reduced by aging, but not differentially changed among the old cognitive groups in the rat PFC^19^. Baclofen-stimulated GTP-binding and GABA_B_ R1 and GABA_B_ R2 proteins were reduced in the PFC of aged rats but these reductions were not associated with spatial learning abilities. In contrast, hippocampal GTP-binding was comparable between young and aged rats but reduced hippocampal GABA_B_ R1 expression was observed in aged rats with spatial learning impairment, indicating that normal aging differentially modulates the expression of GABA_B_Rs between the PFC and hippocampus and these changes have significant effects on signaling efficacy within the PFC but not in the hippocampus^19^. Another study reports reduced GABA_B_ R1 and R2 subunit expression with age in the rat PFC that is associated with increased performance in working memory tests^21^. Furthermore, systematic GABA_B_R antagonist (CGP55845) administration also lead to working memory improvements in aged rats^21^. The current data suggests that the age-related changes in GABA_B_R expression is complex and the functional implications are still not fully understood.

Age-related changes have been observed in the levels of both GAD 65 and GAD 67 isoforms, which are essential in maintaining GABA levels in the brain. We showed significant reductions in GAD65 expression in the STG with age in females^6^. This is in agreement with the previously reported age-related decline in GAD65 expression in the visual cortex in older adults^22^ and rhesus macaque^23^. However, a study demonstrated age-related increase in GAD expression in another cortical region, the middle prefrontal cortex (PFC) in rats^21^. In addition, a GABA binding study in rats has shown significantly reduced binding in the substantia nigra and hypothalamus of aged rat brains^24^. These findings suggest that age-related changes of the GABAergic system are brain region-specific.

There has been an increasing body of evidence showing differences between male and female brains, which has been attributed to genetic and hormonal differences between sexes. Expression of GABA signaling components in the brain is also subject to sex-specific differences^6^. Ovarian hormones have demonstrated the ability to regulate and alter subunit composition of GABA_A_Rs. Progesterone and estradiol alter the expression of GABA_A_R α1 subunit in specific regions of the mouse hippocampus. Furthermore, short-term administration of allopregnalone, a GABA_A_R antagonist, in rats led to an upregulation of the GABA_A_R α4 subunit expression^25^. Changes in hormonal levels (estrogen and progesterone) during the menstrual cycle has been shown to affect GABA levels in the healthy female brain^26,27^. Estradiol has also been shown to have inhibitory actions which can alter the firing of hippocampal neurons^28^. Furthermore, burst in GABA_A_R-dependent inhibitory postsynaptic currents in gonadotropin-releasing hormone neurons are regulated by estrogens^29^. Given the prevalence of such hormonal changes in females during puberty, menstrual cycle, pregnancy and menopause it is plausible that females are more susceptible to changes affecting the GABAergic system. However in regards to males, some evidence suggests that testosterone may cause a decrease in GABA binding sites in the hypothalamus and anterior pituitary gland^30^. These findings suggest that the GABAergic system is affected differently between males and females driven predominantly by hormonal differences.

It is important to consider possible sex- and age-biases, as evidence indicates that these are observed in many neurological conditions such as depression, anxiety, schizophrenia, epilepsy and Alzheimer’s disease (AD)^16,31–37^. The GABAergic system has been directly linked to the pathological changes observed in these conditions. Furthermore, the GABAergic system is a direct target of pharmacological treatments to help treat these conditions.

This study is the first analysis of sex- and age-specific expression of GABAergic signaling components in the Cornu Ammonis (CA1) and Dentate Gyrus (DG) regions of the human hippocampus and the Entorhinal Cortex (ECx). In the current study, we observed that the expression of the GABAergic signaling components are mostly robust to age- and sex-related changes. However, the observed age-related sex-specific alterations in GABAR subunit levels cannot be underestimated as they might significantly influence hippocampal GABAergic neurotransmission and cognitive function.

## Results

**Figure 1:**
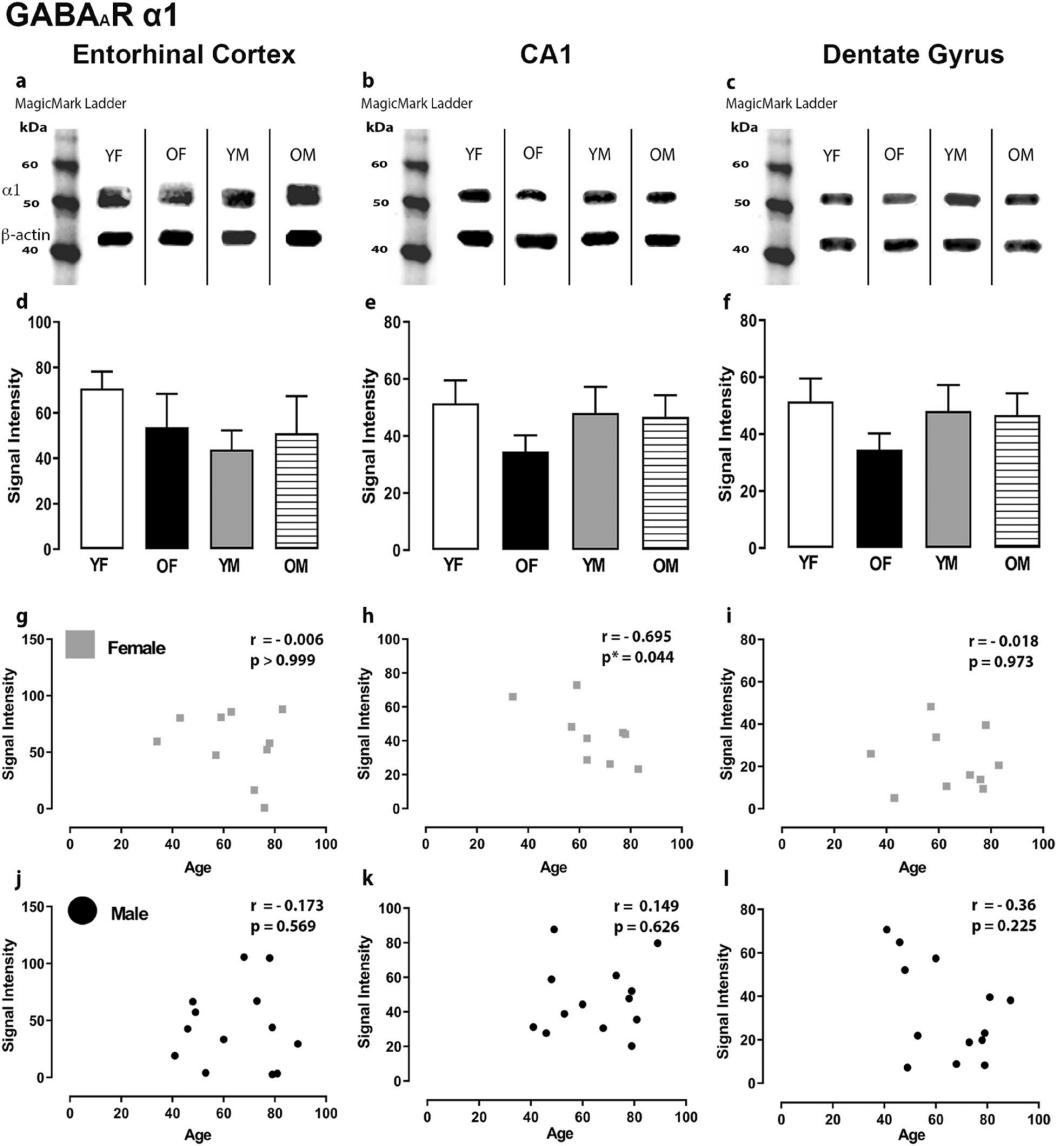
Expression of the GABA_A_R α1 subunit in the entorhinal cortex, hippocampal CA1 region and dentate gyrus. (**a**, **b**, **c**) Representative immunoreactive Western Blot bands from younger female (YF), older female (OF), younger male (YM) and older male (OM) tissue homogenates of the ECx, CA1 and DG regions, following incubation with GABA_A_R α1 antibody. GABA_A_R α1 and corresponding β-actin band (α1 band size- ~ 52 kDa, β-actin band size ~ 42 kDa) are shown. Each lane was loaded with 20 µg of protein. (**d**, **e**, **f**) Signal intensity graphs for each group comparing GABA α1 Western Blot band was measured and normalised to their corresponding β-actin signal for each age group. The data is graphed as mean ± SEM (N = 4–7). (**g**, **h**, **i**, **j**, **k**, **l**) Correlation graphs for males and females plotting the relationship between age and signal intensity of GABA α1 bands.

**Figure 2:**
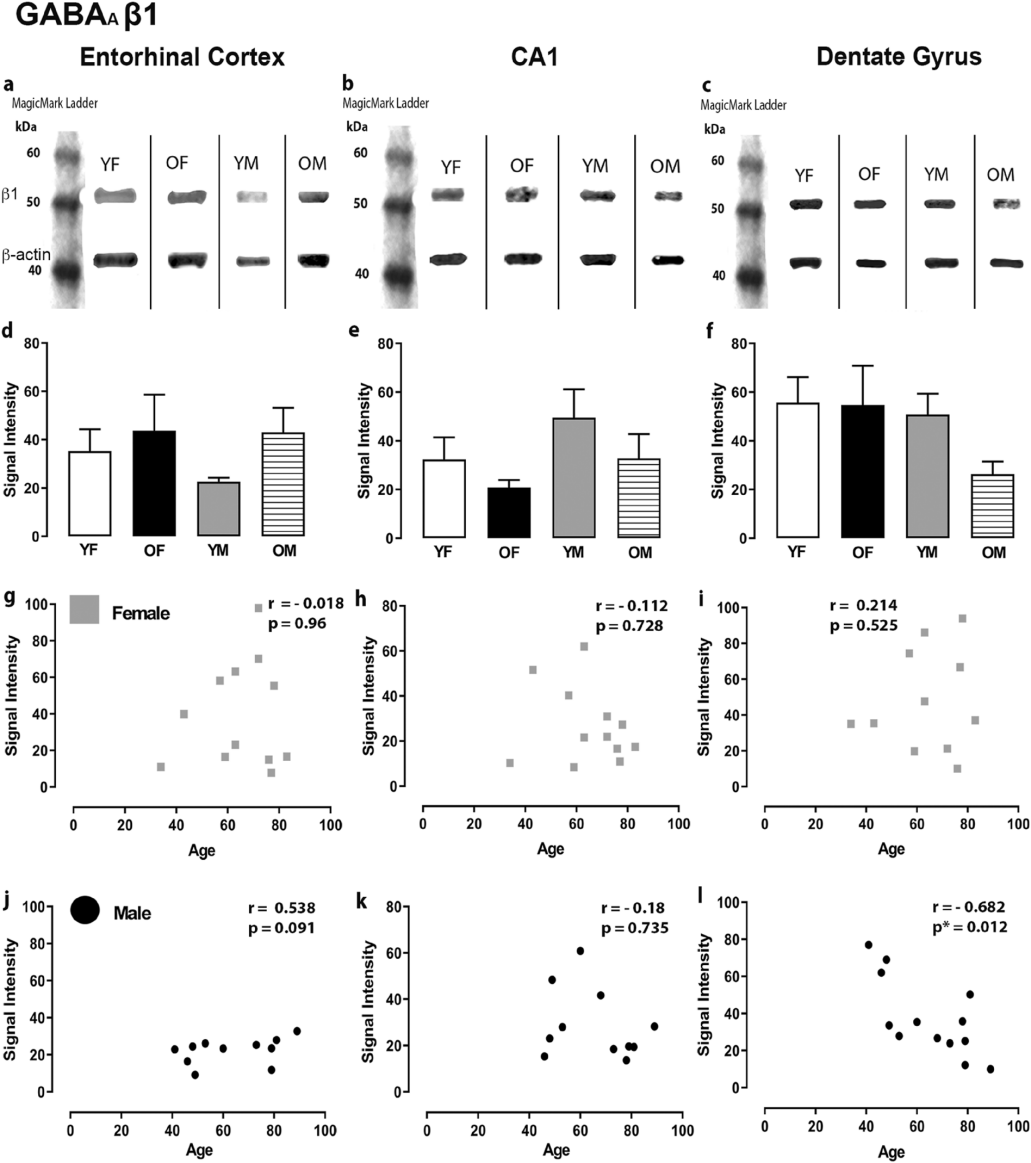
Expression of the GABA_A_R β1 subunit in the entorhinal cortex, hippocampal CA1 region and dentate gyrus. (**a**, **b**, **c**) Representative immunoreactive Western Blot bands from younger female (YF), older female (OF), younger male (YM) and older male (OM) tissue homogenates of the ECx, CA1 and DG regions, following incubation with GABA_A_R β1 antibody. GABA_A_R β1 and corresponding β-actin band (β1 band size- ~ 53 kDa, β-actin band size ~ 42 kDa) are shown. Each lane was loaded with 20 µg of protein. (**d**, **e**, **f**) Signal intensity graphs for each group comparing GABA β1 Western Blot band was measured and normalised to their corresponding β-actin signal for each age group. The data is graphed as mean ± SEM (N = 4–7). (**g**, **h**, **i**, **j**, **k**, **l)** Correlation graphs for males and females plotting the relationship between age and signal intensity of GABA β1 bands (p * ≤ 0.05).

**Figure 3:**
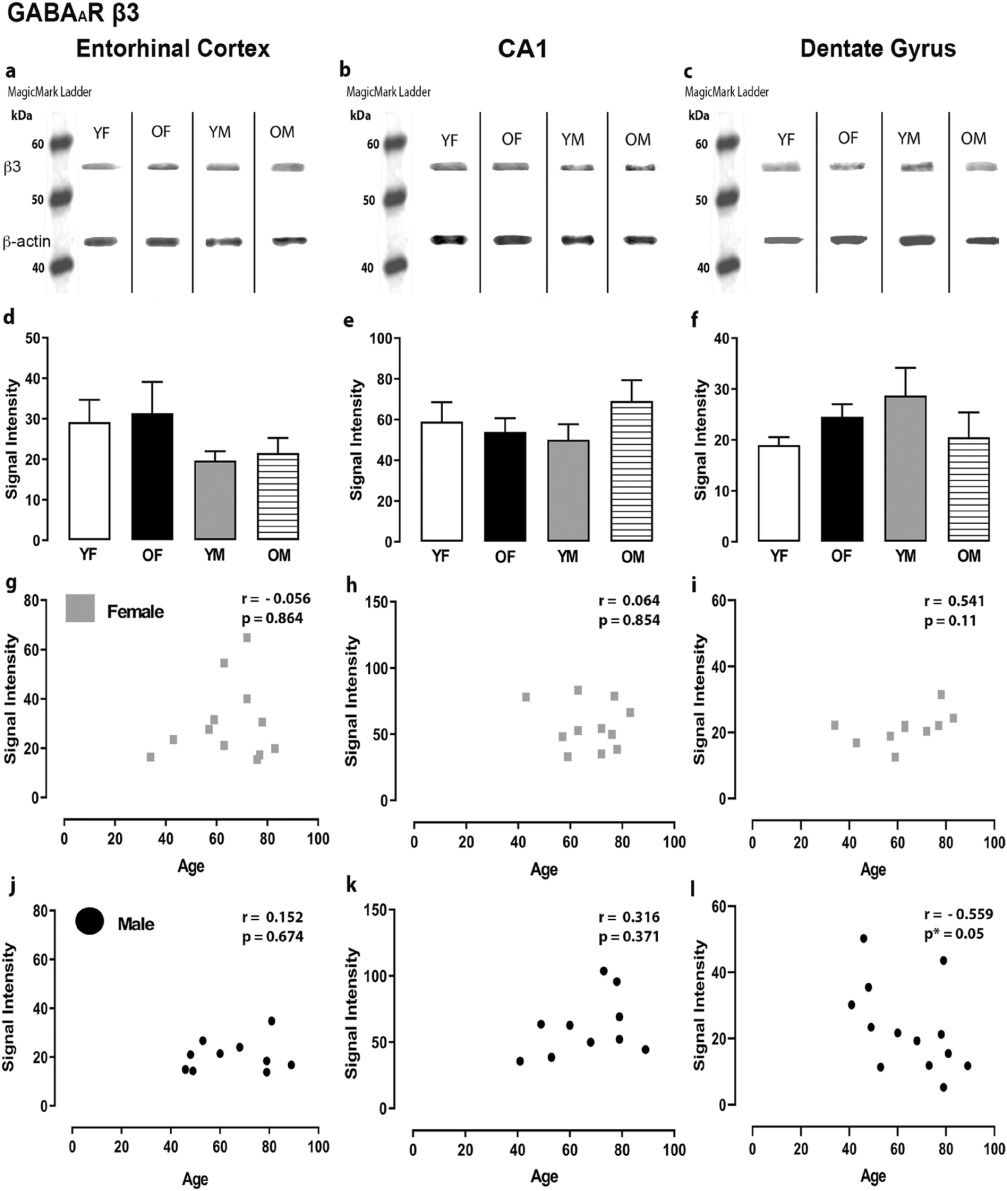
Expression of the GABA_A_R β3 subunit in the entorhinal cortex, hippocampal CA1 region and dentate gyrus. (**a**, **b**, **c**) Representative immunoreactive Western Blot bands from younger female (YF), older female (OF), younger male (YM) and older male (OM) tissue homogenates of the ECx, CA1 and DG regions, following incubation with GABA_A_R β3 antibody. GABA_A_R β3 and corresponding β-actin band (β3 band size- ~ 52 kDa, β-actin band size ~ 42 kDa) are shown. Each lane was loaded with 20 µg of protein. (**d**, **e**, **f**) Signal intensity graphs for each group comparing GABA β3 Western Blot band was measured and normalised to their corresponding β-actin signal for each age group. The data is graphed as mean ± SEM (N = 4–7). (**g**, **h**, **i**, **j**, **k**, **l**) Correlation graphs for males and females plotting the relationship between age and signal intensity of GABA β3 bands (p * ≤ 0.05).

**Figure 4:**
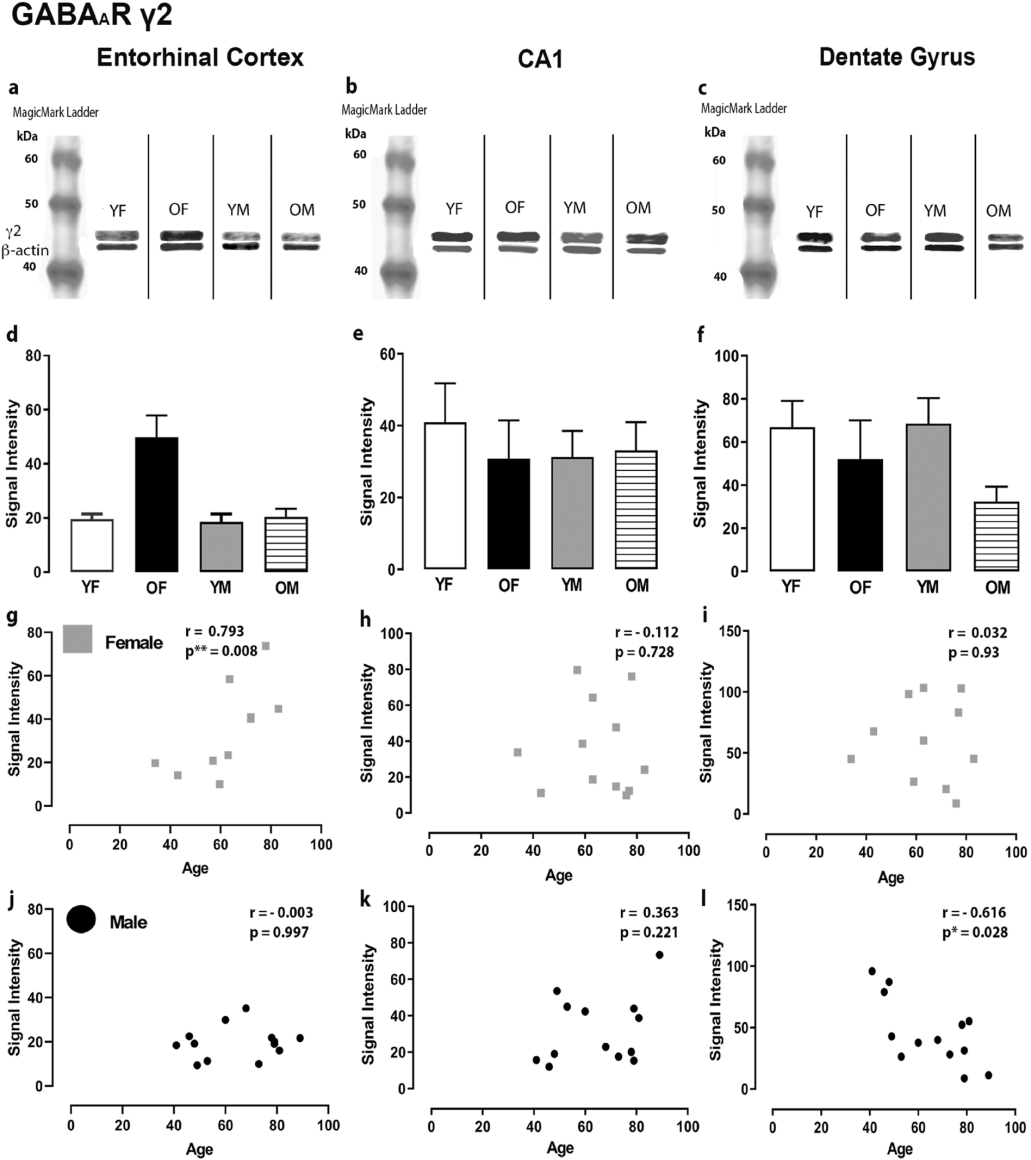
Expression of the GABA_A_R γ2 subunit in the entorhinal cortex, hippocampal CA1 region and dentate gyrus. (**a**, **b**, **c**) Representative immunoreactive Western Blot bands from younger female (YF), older female (OF), younger male (YM) and older male (OM) tissue homogenates of the ECx, CA1 and DG regions, following incubation with GABA_A_R γ2 antibody. GABA_A_R γ2 and corresponding β-actin band (γ2 band size- ~ 44 kDa, β-actin band size ~ 42 kDa) are shown. Each lane was loaded with 20 µg of protein. (**d**, **e**, **f**) Signal intensity graphs for each group comparing GABA_A_R γ2 Western Blot band was measured and normalised to their corresponding β-actin signal for each age group. The data is graphed as mean ± SEM (N = 4–7). (**g**, **h**, **i**, **j**, **k**, **l**) Correlation graphs for males and females plotting the relationship between age and signal intensity of GABA_A_R γ2 bands (p * ≤ 0.001).

**Figure 5:**
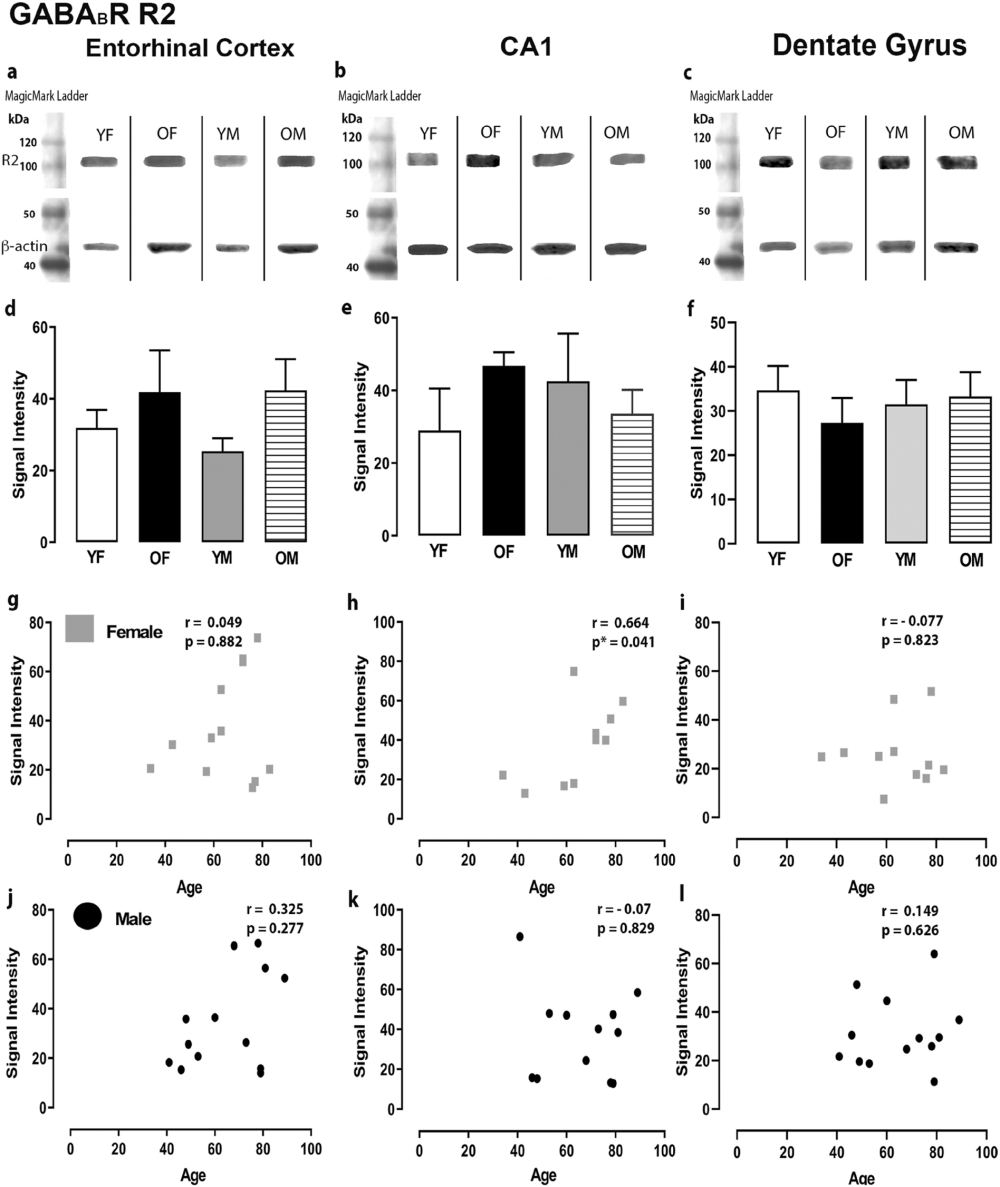
Expression of the GABA_B_R R2 subunit in the entorhinal cortex, hippocampal CA1 region and dentate gyrus. (**a,b,c**) Representative immunoreactive Western Blot bands from younger female (YF), older female (OF), younger male (YM) and older male (OM) tissue homogenates of the ECx, CA1 and DG regions, following incubation with GABABR R2 antibody. GABA_B_R R2 and corresponding β-actin band (R2 band size - ~105kDa, β-actin band size ~ 42 kDa) are shown. Each lane was loaded with 20µg of protein. (**d,e,f**) Signal intensity graphs for each group comparing GABA_B_R R2 Western Blot band was measured and normalised to their corresponding β-actin signal for each age group. The data is graphed as mean ± SEM (N = 4-7). (**g,h,i,j,k,l**) Correlation graphs for males and females plotting the relationship between age and signal intensity of GABA_B_R R2 bands (p * ≤ 0.05).

The expression levels of GABA signaling components, GABA_A_R α1,2,3,5, β1-3, γ2, GABA_B_R R1 and R2 subunits and GAD 65/67 were examined by Western blotting in the ECx, CA1 and DG regions of the human hippocampus. In these brain regions, the GABA_A_R α1,2,3,5, β1-3, γ2, GABA_B_R R1 and R2 subunits and GAD 65/67 were relatively well-preserved during aging. No significant differences were found in protein levels in the ECx, CA1 and DG regions between the younger and older age groups for both sexes (Figs. 1, 2, 3, 4, 5, Suppl Figs. 1–7). However, we observed significant correlations between age and signal intensity for a few GABA signaling components. We detected a significant negative correlation between age and GABA_A_R α1 subunit expression level in the CA1 region of females (Fig. 1h) (r = −0.695, *P* = 0.044). In the DG, there were significant negative correlations between age and expression of GABA_A_R β1 (Fig. 2l) (r = −0.682, *P* = 0.012), β3 (Fig. 3l) (r = −0.559, *P* = 0.05) and γ2 subunits (Fig. 4l) (r = −0.616, *P* = 0.028) for males, respectively. The difference in GABA_A_R γ2 subunit expression level in the ECx between younger females and older females was not statistically significant (Fig. 4d) but showed a trend towards increase in older females. Accordingly, in the ECx we found a significant strong positive correlation between age and expression level of the GABA_A_R γ2 subunit in females (Fig. 4g) (r = 0.793, *P* = 0.008).

A significant positive correlation between age and expression level of GABA_B_R R2 subunit was observed in the CA1 region of females (Fig. 5 h) (r = 0.664, *P* = 0.041). There were no significant age-specific differences in the expression of the GAD 65 (Suppl Fig. 6d,e,f) and GAD 67 enzymes (Suppl Fig. 7d,e,f) between the younger and older age groups. Furthermore, no significant correlations were detected between age and expression level of GAD 65 (Suppl Fig. 6 g-l) or GAD 67 (Suppl Fig. 6 g-l) enzymes in the DG, CA1 region or ECx.

The GABA signaling components examined showed similar expression between females and males and younger and older age groups (Figs. 1, 2, 3, 4, 5, Suppl Figs. 1–7) applying a linear mixed model (Suppl Tables 3–4). This analysis, that takes into consideration the interactions between the brain regions, identified GABA_A_R α1 subunit expression in the CA1 region was significantly different from the DG region (*P* = 0.008); GABA_A_R α3 subunit expression in the ECx region was significantly different from the CA1 region (*P* < 0.001) and the DG (*P* = 0.029); GABA_A_R α5 subunit expression in the ECx region was significantly different from the CA1 region (*P* < 0.001) and the DG (*P* = 0.039), furthermore the GABA_A_R α5 subunit expression in the DG region was significantly different from the CA1 region (*P* = 0.019); GABA_A_R β3 subunit expression in the CA1 region was significantly different from the ECx region (*P* < 0.001) and the DG (*P* < 0.001); GABA_A_R γ2 subunit expression in the CA1 region was significantly different from the ECx region (*P* = 0.039) and the DG (*P* = 0.009), and the GABA_A_R γ2 subunit expression in the DG region was significantly different from the ECx region (*P* < 0.001); GABA_B_R R1 subunit expression in the CA1 region was significantly different from the DG region (*P* < 0.001) (Suppl Tables 3–4).

## Discussion

In this study, we have investigated the effect of age and sex on the expression of GABA_A_R subunits, GABA_B_R subunits, and GABA synthesizing enzymes in the human hippocampus and ECx. We report a significant negative correlation between age and GABA_A_R α1subunit level in the CA1 region for females; a significant negative correlation between age and GABA_A_R β1, β3, and γ2 subunit expression in the DG region for males. In females a significant positive correlation was found between age and GABA_A_R γ2 subunit expression in the ECx and GABA_B_R R2 subunit expression in the CA1 region. However, the results indicate that age and sex do not affect the expression of GAD 65/67. Given the lack of human studies in the literature, the current study provides an important first glimpse into potential age- and sex-related GABA receptor subunit expression changes occurring in the human hippocampus.

In the ECx we found a significant strong positive correlation between age and expression level of the GABA_A_R γ2 subunit in females. The sex-related GABA_A_R γ2 subunit expression increase with age in females might be the result of underlying hormonal differences between sexes. Weiland and Orchinik^27^ showed an increase in GABA_A_R γ2 subunit mRNA levels in the CA1, CA2, and CA3 regions in adult female rats after administration of progesterone via injection. However, in the context of the current study, evidence has shown that there is a decrease in progesterone level with age, with a 30–50% decrease in post-menopausal women and a 35% decrease in estrogen levels^38^. Given that the mean age of the older female group (77.2 ± 3.96 years), the females in the group would be expected to have a reduction of progesterone and estrogen^39^. However, there is no definite evidence that hormonal differences could lead to the sex-specific differences in GABA_A_R γ2 subunit expression observed in this study. A single progesterone treatment might affect the expression of the GABA_A_R γ2 subunit on a different way compared to a long-term reduction in hormonal levels. Additionally, in the in vivo human multiple hormones are acting at any one time.

We also observed a significant strong negative correlation between age and GABA_A_R γ2 subunit expression in the DG of males. In females a significant positive correlation was found between age and GABA_A_R γ2 subunit expression in the ECx, suggesting that these age-related correlations are sex-specific. We found no significant age-related GABA_A_R γ2 subunit level changes in the hippocampus or ECx between the younger and older groups and our recent mouse study shows no age-related alteration when comparing hippocampal (CA1, CA2/3, and DG) γ2 subunit levels in 6 months old (young) and a 21 months (old) male mice^11^. One study found an increase in γ2 subunit expression with age in the rat hippocampus^13^, while others showed an age-related decline in γ2 subunit expression in the mouse hippocampus^14^. However, these studies did not differentiate between hippocampal subregions, sex of the animals, and the rat study examined two age groups only, making it difficult to compare the findings with our results and draw any conclusions about the effect of sex.

Given the role of GABA as the primary inhibitory neurotransmitter in the brain, GABAergic dysfunction has been implicated as one of the factors in epileptic seizure incidences^34,40,41^. GABAergic signaling components are also affected in the epileptic brain, particularly GABA_A_Rs^34,42^. In rat models, treatment with GABA_A_R antagonists such as bicuculline and picrotoxin caused severe motor seizures (Fisher, 1989). Previous studies show the γ2 subunit is widely expressed in the hippocampus and cortical brain areas and is required for the majority of GABA_A_Rs assembly^11,43–46^. Knockout of the GABA_A_R γ2 subunit in mice have shown a reduction in receptor clustering and decreased synaptic function, while γ2 subunit gene disruption showed a near-complete abolishment of all GABA_A_Rs to benzodiazepine site ligands^47^. The higher γ2 subunit levels observed in older females in this study indicate that GABAergic inhibition and effects of benzodiazepines might be more effective in older females in the ECx compared with younger females and both younger and older males. This may be particularly important in seizure therapy, as it has been shown that the ECx is involved in temporal lobe epilepsy (TLE), with TLE patients showing reduced ECx volume^48,49^. The reduced expression of γ2 subunit given the reduced volume and lower expression levels might have more severe implications in males and younger females, however, more research is required in this regard.

We detected a significant negative correlation between age and GABA_A_R α1 subunit level in the CA1 region for females. Hippocampal GABA_A_R α1 subunit expression is high in the CA1 region^11,45,50^, and few rodent studies have shown that this expression stays at the same level^11,14^ or increases^12,13^ with age. However, the expression was not evaluated in females and most studies examined two age groups only. Thus, the results are not comparable to the findings in our study. These findings were also contradicted by studies that found an age-dependent decrease in the α1 subunit expression in the CA1 region of the rhesus monkey and human hippocampus using immunohistochemistry combined with densitometric analysis^51,52^. Previous studies have shown there is a significant loss of GABA_A_R α1 subunit expression in the CA1 and CA2 regions of human hippocampal sections of patients with temporal lobe epilepsy^53,54^. The negative correlation of age and expression of the α1 subunit in the CA1 subregion in females indicates that anti-epileptic drugs might require different dosing for older females, to achieve the best outcomes.

Further significant strong negative correlations were also observed between age and GABA_A_R β1 and β3 subunit levels in the DG region of males. Reduction or elimination of activity at β1 subunit-containing GABA_A_Rs has been shown to increase the efficacy of anxiolytic benzodiazepines in rats^55^. Therefore, age-related decreases in β1 subunit expression in older males might be beneficial for the efficacy and reducing potential side-effects of particular benzodiazepine treatments for anxiety and depression. Previous rat, mouse, and nonhuman primate studies found no significant age-related changes in GABA_A_R β3 subunit expression in the hippocampus^11–13,52^. The α5β3γ2 receptor configuration is particularly high in extra-synaptic sites in the DG^56^ with an important role in cognition and memory, and as a target for benzodiazepine agonists^56,57^. Reduced expression of GABA_A_R β3 subunit in the DG with age in males, as seen in the current study, can affect the receptor configuration, thus reducing GABAergic inhibition and benzodiazepine effectiveness. However, this is not only applicable to benzodiazepines as other drugs also target GABA_A_Rs containing the β3 subunit. An example is loreclezole, an anticonvulsant drug that is a positive allosteric modulator of the GABA_A_R, that is highly selective for receptors containing the GABA_A_R β2 or β3 subunits, unlike classical benzodiazepines which bind to the benzodiazepine binding site between the α and γ subunits of the GABA_A_R^58^. Reduced expression of the GABA_A_R β3 subunit with age can, therefore, affect the therapeutic ability of this and other similar drugs.

Another significant strong positive correlation between age and expression of GABA_B_ R2 subunit was found in the CA1 region of females in our study. The current literature is limited on age- and sex-related changes in the expression of the GABA_B_Rs in the hippocampus. McQuail et al.^19^ found no significant differences in the rat hippocampal GABA_B_R R2 expression and GTP binding. Interestingly, Banuelos et al. (2014) also demonstrated a significant negative correlation between GABA_B_R R2 subunit expression and working memory test scores, while McQuail et al.^19^ found no relationship. The increased expression in GABA_B_R R2 subunits may provide a foundation to understanding the age-related deficits in spatial and working memory, given the findings from studies above indicating increased GABA_B_R R2 subunit expression resulting in a reduced working memory performance^19^. The findings from our current study are in line with the results of Liao et al.^23^ which showed an increase in GABA_B_R R2 subunit expression in the visual cortex of aged rhesus macaque monkeys. However, Pandya et al.^6^ showed no age-related changes in several human cortical areas and cerebellum, and McQuail et al.^19^ and Bañuelos et al.^21^ showed an age-related decrease in GABA_B_R R2 expression in the rat PFC. These studies demonstrate that age-related GABA_B_R R2 subunit expression changes are brain region-specific.

It is known that the hippocampus is involved in spatial learning, memory consolidation, and memory transfer, however, the hippocampus and the GABAergic system are also implicated in neurological disease such as epilepsy, anxiety, depression, and AD^16,34,59,60^. Women are more likely to develop anxiety, depression and dementias such as AD than men^36,60–64^. Importantly, the observed sex-specific expression of GABA_A_R subunits related to anxiety (β2, β3 and γ2) and memory (α1 and α5) might be linked to increased vulnerability to these and other neurological diseases and neurodegenerative conditions. GABA_A_R subunit expression changes have been implicated in AD^16,45,65^. For a long time, the literature has indicated that the GABAergic system remains unperturbed in AD, however, evidence indicates that there are reductions of GABA currents in human cortical cells from AD brains^66^, as well as GABAergic nerve terminal damage and reduction in GABA uptake in the AD brain^67^. Given that the characteristic symptom of AD patients is memory loss, evidence shows the hippocampus is affected severely in AD and also shows age-related molecular and cellular changes^8–10^. Studies have shown that the subunit density of γ2 subunit expression was preserved^68^ and α1 subunit immunolabeling was increased in human AD hippocampal tissue^69^. We reported significant increases in the GABA_A_R α1, α2, α5, β2, and γ2 subunit expression in the different layers of CA1-3 and DG regions in the AD human hippocampus^45^. This is an overall trend except for the α1 and α2 subunits that show decreased expression of the CA1 subregion in the stratum radiatum and pyramidale, respectively^45,60^. In the current study, the expression of GABA_A_R subunit α1 in the CA1 and β1, β3 in the DG in females and males respectively, all show significant negative trends of expression with age. This indicates an opposite effect of normal aging on GABA_A_R subunit expression compared to AD. There is increasing evidence of remodeling of the GABAergic, cholinergic, and glutamatergic neurotransmitter systems in AD leading to disruption of the excitatory/inhibitory balance^16,69–72^. Alterations in glutamate receptor and transporter expression in the hippocampus have also been observed, contributing to glutamate-mediated excitotoxicity^65,71,72–76,87^. The imbalance is further driven by neuronal cell death observed in AD^10,65,77^. Given the findings of these studies, there is some credence to the idea that surviving neurons in the AD hippocampus increase GABA_A_R subunit synthesis to help maintain the inhibitory circuit in the hippocampus, whereas in normal aging, there is a decrease in subunit synthesis.

The results from this study observed an age-related trend toward a decrease in α5 subunit expression with age in the ECx for females that did not reach statistical significance. Findings from a rat study showed a moderate α5 subunit decrease in the hippocampus during aging^14^ but other studies did not detect age-related changes in the mouse and rat hippocampus^11,13^. In AD, increased expression of the GABA_A_R α5 subunit is found in the hippocampal CA1 subregion and subiculum of AD cases compared to healthy controls^45,50,60^. This is in line with findings showing mild memory and cognitive impairment in normal aging with increased GABA_A_R α5 subunit expression^78^. Therefore, it is plausible that the increased GABA_A_R α5 subunit expression is linked to negative cognitive and memory impairments in age-related disease conditions^79^. There have been particular partial inverse agonists, such as S-8510 and α5Ia which appear to have positive effects in reducing memory impairment in rodent lesion and AD models^78–82^. Therefore, an age-related decrease in α5 subunit expression might be compensatory, as it may contribute to maintaining a normal excitatory/inhibitory balance, long-term potentiation, and cognition.

There was no significant age- or sex-specific differences in the expression of GABA_A_R α2 and α3 subunits in the current study. Rodent studies have shown that both the GABA_A_R α2 and α3 subunits decrease with age in the somatosensory and visual cortices^14,22,83,84^. However, a comprehensive mouse study by Palpagama et al.^11^ did not find any age-related expression changes of the aforementioned subunits, which is in agreement with the findings of the current human study. In the human STG GABA_A_R α3 subunit expression is significantly lower in older males^6^ but the subunit levels in most other cortical regions are not affected by age. Given that age-related decreases of the α2 and α3 subunits was only observed in sensory cortices, this could indicate that age-related visual and auditory impairment in the periphery might drive these changes.

Western blotting provides a robust quantitative analysis, but the limitation of this technique is that we were not able to examine the variations in expression across different cell types of neural circuits within hippocampal subregions and ECx. Further studies are required to examine such differences within individual cell types as these could have a significant functional consequence in terms of network activity in these brain regions. These studies will also help to validate our findings. This is important as there is a possibility of false positive and negative errors. While we applied strict case selection criteria and tissue processing and experiments were performed at the highest possible standards the variability of data is relatively high. The study would certainly benefit from more samples and samples that are more evenly spaced by age. However, the availability of human tissue is very limited, therefore minimizing the variance is challenging. In addition, further studies will be required to understand the functional implications of these age-related and sex-specific GABA_A_R subunit expression changes.

## Conclusions

The current knowledge of GABAergic age-related and sex-specific differences across the hippocampus and surrounding cortices is limited. Our study shows that the GABAergic system in the hippocampus and ECx is relatively robust to age-related changes. However, the observed sex-specific negative correlation of GABA_A_ α1, β1, β3, and γ2 subunit expression with age, particularly in the DG and CA1 subregion, and the positive correlation of the γ2 and GABA_B_ R2 subunit expression with age in the ECx and CA1 subregion, respectively, might significantly influence the function of the receptors and affect GABAergic inhibition within the human hippocampus. As discussed above the GABAergic system is implicated in the development and progression of several neurological disorders, therefore important to consider sex-and age-specific differences in the expression of GABA signaling components when designing new therapeutics or improving current treatments. Age-related functional changes such as cognitive decline, depression, and increased risk of neurodegenerative disease are becoming more of a pressing issue given the growing elderly population. Understanding the mechanisms involved in aging of a critically important neurotransmitter system, the GABAergic system, will help provide better understanding of pathological changes that might be accelerated by age. Further research will be important to shed light on the implications of age- and sex-related GABAergic alterations in disease conditions, to design improved and more effective personalized treatments.

## Methods

### Human brain tissue preparation and neuropathological analysis

The study was conducted at the University of Auckland, Centre for Brain Research. The tissue was acquired through a donation program to the Neurological Foundation Human Brain Bank. The procedures were approved by the University of Auckland Human Participants’ Ethics Committee. All experiments were performed in accordance with relevant guidelines and regulations. Processing of tissue followed the procedure described previously^85^. The brain was dissected to separate the hemispheres, with the left hemisphere cut into anatomical blocks and freshly frozen and stored at −80 °C. Standard sections, including the middle frontal-, middle temporal-, cingulate gyrus, hippocampus, caudate nucleus, substantia nigra, locus coeruleus, cerebellum from all cases were examined by a neuropathologist. All cases included in this study had no history of any primary neurodegenerative, psychiatric disorder, neurological disease abnormalities, or excessive alcohol consumption (Suppl Table 1). None of our records show that that any of the female cases were on hormone replacement therapy. The cases were sorted into four groups-younger female (YF-53.2 years ± 11.9; N = 6), older female (OF 77.2 years ± 3.96; N = 6), younger male (YM-49.5 years ± 6.5; N = 6), older male (OM-78.14 years ± 6.47; N = 7). All effort was made to have the largest possible age gap between the younger and older age groups.

### Western blotting

The human tissue blocks were cut using a cryostat (CM3050, Leica Microsystems, Germany) at 60-µm thickness and collected on glass slides. Hippocampal areas of interest-DG, CA1 and ECx-were collected with a blade into sterile 1.7 ml tubes for each area of interest. The tissue was homogenised in a buffer containing 0.5 M Tris, 100 mM EDTA, 4% SDS, pH 6.8, supplemented with 100 mM phenylmethanesulfonyl fluoride (Sigma, St. Louis, MO, USA) and 0.5 mm glass beads (Mo-Bio Laboratories, Solano Beach, CA, USA) in a Mini Bullet Blender Tissue Homogenizer (Next Advance, Inc., NY, USA) at speed 8 for 8 min. The homogenates were incubated for 1 h on ice, centrifuged at 10621 g for 10 min. The resulting supernatant collected and stored at −20 °C. The protein concentration of each sample was determined using detergent-compatible protein assay (DC Protein Assay, 500–0116, Bio-Rad, Hercules, CA, USA) as per manufacturer instructions.

Protein samples for each case were numbered from 1 to 25 and randomized. The experimenter was blinded to avoid any potential bias during the experiment, image acquisition and analysis.

Twenty μg of each protein extract was run on a gradient polyacrylamide electrophoresis gel (NU PAGE 4–12% BT 1.5, NP0336BOX; Life Technologies, Carlsbad, CA, USA) and then blotted using the Thermofisher XCell Blot Module (Thermofisher, CA, USA), which was then transferred onto nitrocellulose membranes (Amersham-Protran, GE Healthcare, Germany) for immunolabeling. The gels were also loaded with three molecular weight ladders to verify labeled band sizes: MagicMark, SeeBlue and Molecular Weight (Invitrogen, CA, USA). Membranes were blocked with Odyssey blocking buffer (LI-COR Biosciences, NE, USA) at room temperature for 30 min, followed by incubation with primary antibodies (Suppl Table 2) in 5% BSA-Tris-Buffered saline (TBS) pH 7.6, 0.1% Tween (TBST) at 4 °C for 24 h. The membranes were then washed 3 × 10 min in TBST and incubated with the appropriate IRDye (1:10,000, goat anti-rabbit IRDye 680RD, 926–68,071, RRID: AB_10956166: goat anti-mouse IRDye 800CW, 926–32,214, RRID: AB_621846: LI-COR Biosciences, NE, USA) secondary antibody for 1 h at room temperature. Membranes were washed and scanned on an Odyssey Infrared Imaging System (LI-COR Biosciences, NE, USA). Detection of the immunofluorescence signal was carried out at the 680 nm and 800 nm spectrum. No data points were excluded but few bands were not included in analysis due to technical problems, such as problems related to loading, running, background and damage of the gels or membranes. Representative bands from blots were cropped from different parts of the same gel (labeled with asterisk), for full length blots see Supplementary Information.

### Analysis

To measure the signal intensities of each sample band, the analysis was conducted using ImageJ software (National Institutes of Health, USA). Signal intensity of each sample was normalised to β-actin. The logarithm of the protein expression was analysed per protein using a linear mixed model with all interactions between Region, Gender and AgeClass as fixed terms and person-id as a random term. The analysis was performed in R, version 4.0.3. (https://www.r-project.org), using package lme4^86^. Subsequent residual analysis was done using the package DHARMa (https://CRAN.R-project.org/package=DHARMa. Correlation analysis was performed using a Spearman’s test in Prism (version 8; GraphPad Software) with a value of *P* ≤ 0.05 considered significant. Data in all figures was expressed as mean ± SEM. Adobe Photoshop CC 2021 (Adobe Systems Software) was used to prepare the figures.

### Ethics approval

All procedures were approved by the University of Auckland Human Participants’ Ethics Committee (Approval number: 001654).

## Supplementary Information

Supplementary Information.

## Data Availability

The datasets used and/or analysed during the current study are available from the corresponding author on reasonable request.
