# Supplementary material for: The effect of age and sex on the expression of GABA signaling components in the human hippocampus and entorhinal cortex
Source: Sci Rep. 2021 Nov 2;11:21470. doi: 10.1038/s41598-021-00792-8 (PMC8563768; doi:10.1038/s41598-021-00792-8)

Supplementary Tables and Figures

The effect of age and sex on the expression of GABA signaling components in the human hippocampus and entorhinal cortex

Jayarjun Ethiraj[1], Thulani Hansika Palpagama[1], Clinton Turner[1,2], Bert van der Werf[3], Henry John Waldvogel[1], Richard Lewis Maxwell Faull[1], Andrea Kwakowsky[1*].

[1]*Centre for Brain Research, Department of Anatomy and Medical Imaging, Faculty of Medical and Health Sciences, School of Medical Sciences, University of Auckland, Auckland, New Zealand*

[2]*Department of Anatomical Pathology, LabPlus, Auckland City Hospital, Auckland, New Zealand*

[3]*Department of Epidemiology and Biostatistics, Faculty of Medical and Health Sciences, School of Population Health, University of Auckland, Auckland, New Zealand*

Corresponding author: Andrea Kwakowsky, PhD

Centre for Brain Research, Department of Anatomy and Medical Imaging, Faculty of Medical and Health Sciences, University of Auckland, Auckland, New Zealand

E-mail: a.kwakowsky@auckland.ac.nz

Supplementary Table 1. Cases used in this study

Case	*Age (years)*	*Gender*	*Post Mortem Delay (hrs)*	*Cause of Death*	*Classification*
H110	83	F	6.5	Pulmonary Embolism	OF
H111	46	M	10	Coronary Artery Disease	YM
H112	79	M	8	Bleeding Stomach Ulcer	OM
H113	68	M	19	Coronary Artery Disease	OM
H122	72	F	9	Emphysema	OF
H123	78	M	7.5	Aortic Aneurysm	OM
H124	49	M	13	Ischemic Heart Disease	YM
H127	59	F	21	Pulmonary Embolism	YF
H128	34	F	18.5	Myocardial Infarction	YF
H129	48	M	12	Pulmonary Embolism	YM
H131	73	M	13	Ischemic Heart Disease	OM
H132	63	F	10	Ruptured Aorta	YF
H137	77	F	12	Coronary Atherosclerosis	OF
H142	41	M	16	Heart Disease	YM
H152	79	M	18	Congestive Heart Failure	OM
H159	53	M	16.5	Ischemic Heart Disease	YM
H165	43	F	24	Nitrogen Poisoning	YF
H169	81	M	24	Carbon Monoxide Poisoning	OM
H170	60	M	17	Ischemic Heart Disease	YM
H181	78	F	20	Aortic Aneurysm	OF
H190	72	F	19	Ruptured Myocardial Infarction	OF
H230	57	F	32	Carcinomatosis Renal	YF
H238	63	F	16	Dissecting Aortic Aneurysm	YF
H241	76	F	12	Metastatic Bowel Cancer	OF
H246	89	M	17	Myocardial Infarction	OM

Supplementary Table 2. Primary antibodies used in this study

	Antigen	Host Animal	Company	Catalogue Number	Concentration	Immunogen	Specificity	Reference
GABA_A Receptor Subunits	***α1***	Mouse	Gift from JM Fritschy and H Mohler	Bd-24	1:1000	Peptide QPSQDELKDNTTVFTR	α1 subunit	Fritschy and Mohler (1995)
	α2	Rabbit	Alomone	AGA-002	1:200	Peptide (C)TPEPNKKPENKPA	α2 subunit	Pandya *et al.* (2019) Kwakowsky *et al.* (2018c)
	α3	Rabbit	Alomone	AGA-003	1:200	Peptide QGESRRQEPGDFVKQ	α3 subunit	Seo and Leitch (2014) Pandya *et al.* (2019)
	α5	Rabbit	Thermofisher	PA5-3116	1:200	Amino acids 142 and 379	α5 subunit	Pandya *et al.* (2019)
	β1	Rabbit	R&D Systems	PPS 030B	1:250	Fusion protein from the cytosolic loop of the rat $GABA_AR$ β1 subunit, C-Terminus	β1 Subunit	Kwakowsky *et al.* (2018c)
	β2	Rabbit	Novus	NB-51214	1:250	Synthetic peptide, corresponding to amino acids 400-450 of human $GABA_AR$ β2	β2 subunit	Kelley *et al.* (2013)
	β3	Mouse	Fritschy	BD-17	1:500	Purified GABA/benzodiazepine receptor from bovine cortex	β2 & β3 subunit	Fritschy and Mohler (1995)

		γ2	Rabbit	Synaptic Systems	224 003	1:200	Synthetic peptide corresponding to AA 39 to 67 from mouse $GABA_AR$ γ2	γ2 subunit	Wu *et al.* (2018)
GABA$_B$ Receptor Subunits		***BR1***	Goat	Santa Cruz	SC-166408	1:200	Amino acids 929-958 at the C-terminus of $GABA_BR$ R1 of rat origin	BR1 subunit	Konermann et al. (2016)
		BR2	Mouse	NeuroMab	75-124	1:200	Fusion protein amino acids 862-913 (cytoplasmic C-terminus) of human $GABA_BR$ R2	BR2 subunit	Workman *et al.* (2015)
GAD Enzymes		***GAD65/67***	Rabbit	Sigma-Aldrich	G5163	1:500	Synthetic peptide corresponding to the C-terminal region of human GAD 67 (amino acids 579-594). Sequence is identical in human GAD 65 (amino acids 570-585)	GAD 65 & 67 enzyme	Levesque and Parent (2005)
β - Actin		***β - Actin***	Mouse	Abcam	ab6276	1:1000	Synthetic peptide conjugated to KLH derived from within residues 1 - 100 of human beta actin	β- actin	Hu *et al.* (2018)
		β - Actin	Rabbit	Abcam	ab8227	1:1000	Synthetic peptide within human beta actin amino acid 1-100	β - actin	Wang *et al.* (2016)

Supplementary Table 3. Linear mixed model - ANOVA results

Protein	SourceOfVariation	Sum Sq	Mean Sq	NumDF	DenDF	F value	Pr(>F)
Alpha 1	Region	5.41	2.71	2.00	34.94	3.77	0.0329
	Gender	0.00	0.00	1.00	16.82	0.00	0.9575
	AgeClass	1.76	1.76	1.00	16.82	2.46	0.1355
	Region:Gender	0.88	0.44	2.00	34.94	0.61	0.5470
	Region:AgeClass	0.44	0.22	2.00	34.94	0.31	0.7382
	Gender:AgeClass	0.55	0.55	1.00	16.82	0.77	0.3917
	Region:Gender:AgeClass	1.70	0.85	2.00	34.94	1.18	0.3186
Alpha 2	Region	0.32	0.16	2.00	35.59	1.08	0.3507
	Gender	0.03	0.03	1.00	19.94	0.19	0.6693
	AgeClass	0.01	0.01	1.00	19.94	0.04	0.8531
	Region:Gender	0.36	0.18	2.00	35.59	1.20	0.3133
	Region:AgeClass	0.24	0.12	2.00	35.59	0.81	0.4528
	Gender:AgeClass	0.02	0.02	1.00	19.94	0.17	0.6875
	Region:Gender:AgeClass	0.19	0.10	2.00	35.59	0.65	0.5289
Alpha 3	Region	2.51	1.26	2.00	38.00	7.57	0.0017
	Gender	0.03	0.03	1.00	19.00	0.15	0.6995
	AgeClass	0.00	0.00	1.00	19.00	0.00	0.9504
	Region:Gender	0.46	0.23	2.00	38.00	1.39	0.2605
	Region:AgeClass	0.18	0.09	2.00	38.00	0.54	0.5891
	Gender:AgeClass	0.00	0.00	1.00	19.00	0.01	0.9129
	Region:Gender:AgeClass	0.29	0.15	2.00	38.00	0.88	0.4239
Alpha 5	Region	4.75	2.38	2.00	38.45	10.72	0.0002
	Gender	0.02	0.02	1.00	19.76	0.10	0.7499
	AgeClass	0.72	0.72	1.00	19.76	3.25	0.0868
	Region:Gender	0.77	0.39	2.00	38.45	1.74	0.1889
	Region:AgeClass	0.02	0.01	2.00	38.45	0.04	0.9608
	Gender:AgeClass	0.10	0.10	1.00	19.76	0.43	0.5197
	Region:Gender:AgeClass	0.49	0.24	2.00	38.45	1.10	0.3435
Beta 1	Region	0.07	0.03	2.00	42.00	0.09	0.9139
	Gender	0.00	0.00	1.00	21.00	0.00	0.9876
	AgeClass	0.23	0.23	1.00	21.00	0.62	0.4399
	Region:Gender	1.17	0.59	2.00	42.00	1.55	0.2245
	Region:AgeClass	1.73	0.87	2.00	42.00	2.29	0.1138
	Gender:AgeClass	0.00	0.00	1.00	21.00	0.00	0.9886
	Region:Gender:AgeClass	0.63	0.31	2.00	42.00	0.83	0.4425
Beta 2	Region	0.01	0.00	2.00	41.77	0.01	0.9894
	Gender	0.01	0.01	1.00	21.13	0.04	0.8525
	AgeClass	0.91	0.91	1.00	21.13	2.42	0.1343
	Region:Gender	0.26	0.13	2.00	41.77	0.35	0.7095
	Region:AgeClass	0.02	0.01	2.00	41.77	0.03	0.9680
	Gender:AgeClass	0.08	0.08	1.00	21.13	0.22	0.6436
	Region:Gender:AgeClass	0.77	0.39	2.00	41.77	1.03	0.3668
Beta 3	Region	12.97	6.49	2.00	38.05	35.50	0.0000
	Gender	0.04	0.04	1.00	20.10	0.24	0.6313
	AgeClass	0.01	0.01	1.00	20.10	0.03	0.8610
	Region:Gender	0.59	0.29	2.00	38.05	1.61	0.2139
	Region:AgeClass	0.14	0.07	2.00	38.05	0.38	0.6895
	Gender:AgeClass	0.04	0.04	1.00	20.10	0.22	0.6472
	Region:Gender:AgeClass	0.93	0.47	2.00	38.05	2.55	0.0917
Gamma 2	Region	7.55	3.77	2.00	41.19	10.83	0.0002
	Gender	0.13	0.13	1.00	20.86	0.37	0.5478
	AgeClass	0.27	0.27	1.00	20.86	0.76	0.3931
	Region:Gender	0.18	0.09	2.00	41.19	0.25	0.7791
	Region:AgeClass	1.70	0.85	2.00	41.19	2.44	0.0999
	Gender:AgeClass	0.00	0.00	1.00	20.86	0.00	0.9703
	Region:Gender:AgeClass	0.41	0.20	2.00	41.19	0.59	0.5614
BR1	Region	4.62	4.62	1.00	13.33	39.48	0.0000
	Gender	0.00	0.00	1.00	16.83	0.04	0.8469
	AgeClass	0.00	0.00	1.00	16.83	0.03	0.8585
	Region:Gender	0.02	0.02	1.00	13.33	0.16	0.6985
	Region:AgeClass	0.05	0.05	1.00	13.33	0.41	0.5313
	Gender:AgeClass	0.25	0.25	1.00	16.83	2.14	0.1620
	Region:Gender:AgeClass	0.20	0.20	1.00	13.33	1.68	0.2171
BR2	Region	0.58	0.29	2.00	40.59	0.98	0.3831
	Gender	0.02	0.02	1.00	21.52	0.05	0.8193
	AgeClass	0.35	0.35	1.00	21.52	1.17	0.2918
	Region:Gender	0.23	0.12	2.00	40.59	0.39	0.6767
	Region:AgeClass	0.30	0.15	2.00	40.59	0.51	0.6059
	Gender:AgeClass	0.10	0.10	1.00	21.52	0.35	0.5612
	Region:Gender:AgeClass	1.01	0.51	2.00	40.59	1.71	0.1941
GAD65	Region	0.49	0.24	2.00	34.80	1.53	0.2314
	Gender	0.01	0.01	1.00	17.81	0.09	0.7657
	AgeClass	0.12	0.12	1.00	17.81	0.78	0.3902
	Region:Gender	0.21	0.11	2.00	34.80	0.67	0.5190
	Region:AgeClass	0.55	0.27	2.00	34.80	1.73	0.1925
	Gender:AgeClass	0.01	0.01	1.00	17.81	0.06	0.8099
	Region:Gender:AgeClass	0.46	0.23	2.00	34.80	1.45	0.2478
GAD67	Region	0.11	0.06	2.00	35.12	0.47	0.6316
	Gender	0.00	0.00	1.00	18.07	0.01	0.9306
	AgeClass	0.01	0.01	1.00	18.07	0.09	0.7622
	Region:Gender	0.06	0.03	2.00	35.12	0.23	0.7923
	Region:AgeClass	0.07	0.04	2.00	35.12	0.28	0.7540
	Gender:AgeClass	0.01	0.01	1.00	18.07	0.08	0.7797
	Region:Gender:AgeClass	0.56	0.28	2.00	35.12	2.26	0.1193

Supplementary Table 4. Linear mixed model - P-values for regions, the marginal estimates are on the logarithmic scale

Protein	Region	estimate	lower.95	upper.95	P-value.CA1	P-value.DG	P-value.ECx
Alpha 1	CA1	3.68	3.27	4.09	1.0000	0.0077	0.4008
Alpha 1	DG	3.00	2.61	3.39	0.0077	1.0000	0.0642
Alpha 1	ECx	3.46	3.06	3.86	0.4008	0.0642	1.0000
Alpha 2	CA1	3.49	3.30	3.69	1.0000	0.2204	0.1877
Alpha 2	DG	3.34	3.14	3.55	0.2204	1.0000	0.9693
Alpha 2	ECx	3.34	3.15	3.53	0.1877	0.9693	1.0000
Alpha 3	CA1	3.96	3.77	4.15	1.0000	0.0904	0.0001
Alpha 3	DG	3.75	3.56	3.94	0.0904	1.0000	0.0288
Alpha 3	Ecx	3.49	3.30	3.68	0.0001	0.0288	1.0000
Alpha 5	CA1	3.78	3.58	3.98	1.0000	0.0188	3.6575E-06
Alpha 5	DG	3.44	3.22	3.66	0.0188	1.0000	0.0393
Alpha 5	ECx	3.15	2.95	3.34	3.6575E-06	0.0393	1.0000
Beta 1	CA1	3.27	3.00	3.55	1.0000	0.6750	0.8797
Beta 1	DG	3.35	3.07	3.62	0.6750	1.0000	0.7887
Beta 1	ECx	3.30	3.02	3.58	0.8797	0.7887	1.0000
Beta 2	CA1	3.57	3.32	3.82	1.0000	0.9922	0.8952
Beta 2	DG	3.57	3.31	3.82	0.9922	1.0000	0.9043
Beta 2	ECx	3.55	3.30	3.80	0.8952	0.9043	1.0000
Beta 3	CA1	4.01	3.82	4.20	1.0000	1.77636E-15	6.22862E-11
Beta 3	DG	2.98	2.80	3.16	1.7763E-15	1.0000	0.1877
Beta 3	ECx	3.15	2.96	3.33	6.2286E-11	0.1877	1.0000
Gamma 2	CA1	3.32	3.04	3.61	1.0000	0.0090	0.0389
Gamma 2	DG	3.77	3.48	4.06	0.0090	1.0000	3.37725E-06
Gamma 2	ECx	2.98	2.69	3.26	0.0389	3.37725E-06	1.0000
BR1	CA1	3.64	3.43	3.85	1.0000	3.3077E-10	
BR1	DG	2.89	2.66	3.11	3.3077E-10	1.0000	
BR2	CA1	3.46	3.22	3.71	1.0000	0.1853	0.7515
BR2	DG	3.25	3.01	3.48	0.1853	1.0000	0.2922
BR2	ECx	3.41	3.18	3.64	0.7515	0.2922	1.0000
GAD65	CA1	3.56	3.36	3.77	1.0000	0.1345	0.1145
GAD65	DG	3.37	3.19	3.55	0.1345	1.0000	0.9143
GAD65	ECx	3.36	3.17	3.55	0.1145	0.9143	1.0000
GAD67	CA1	3.38	3.19	3.56	1.0000	0.4688	0.3508
GAD67	DG	3.30	3.13	3.47	0.4688	1.0000	0.8244
GAD67	ECx	3.27	3.11	3.44	0.3508	0.8244	1.0000

GABA$_A$R α2

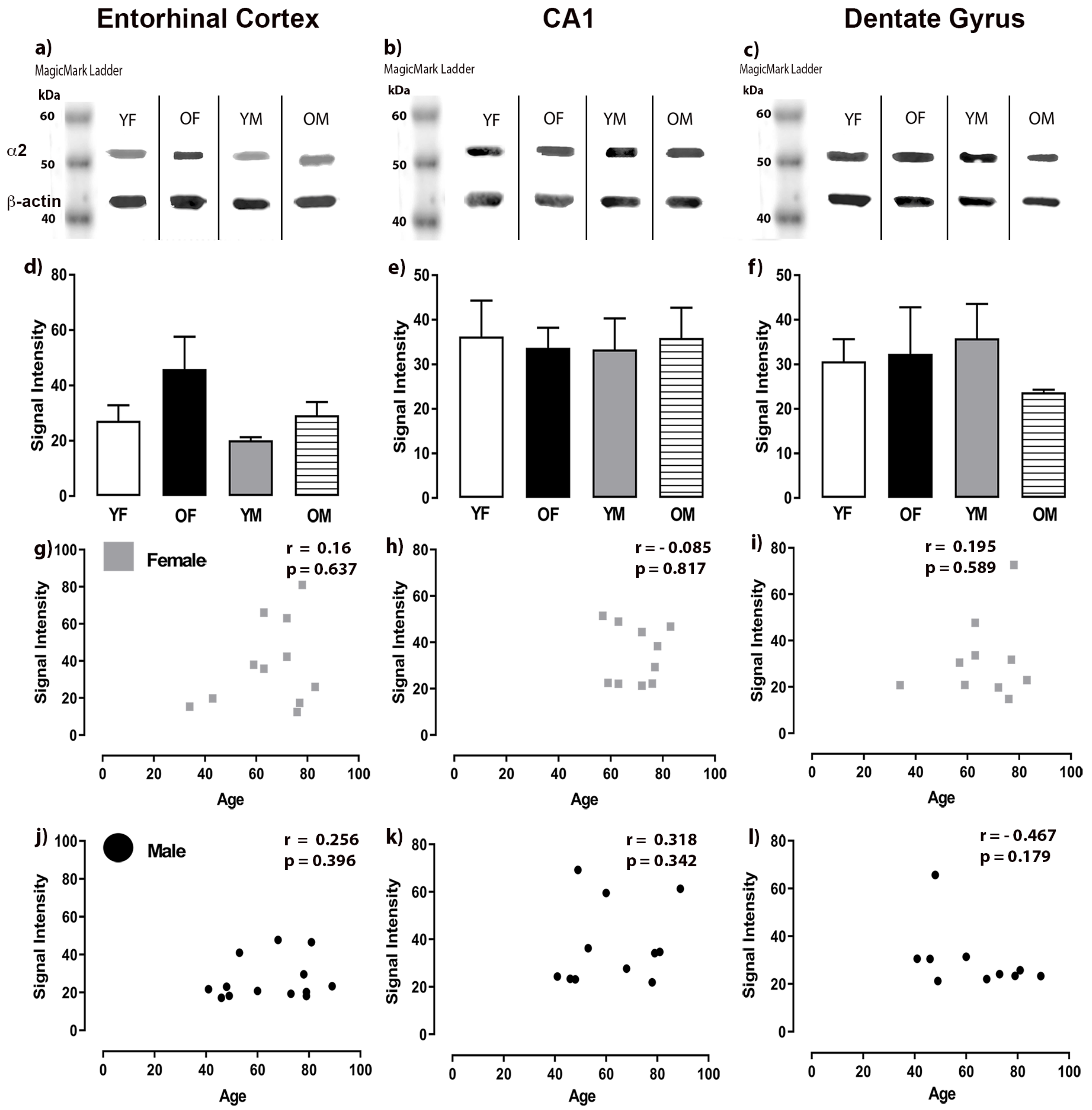

Supplementary Figure 1: Expression of the GABA$_A$R α2 subunit in the entorhinal cortex, hippocampal CA1 region and dentate gyrus. **a,b,c)** Representative immunoreactive Western Blot bands from younger female (YF), older female (OF), younger male (YM) and older male (OM) tissue homogenates of the ECx, CA1 and DG regions, following incubation with GABA$_A$R α2 antibody. GABA$_A$R α2 and corresponding β-actin band (α2 band size - ~51kDa, β-actin band size ~ 42 kDa) are shown. Each lane was loaded with 20µg of protein. **d,e,f)** Signal intensity graphs for each group comparing GABA α2 Western Blot band was measured and normalised to their corresponding β-actin signal for each age group. The data is graphed as mean ± SEM (N = 4-7). **g,h,i,j,k,l)** Correlation graphs for males and females plotting the relationship between age and signal intensity of GABA α2 bands.

GABA$_A$R α3

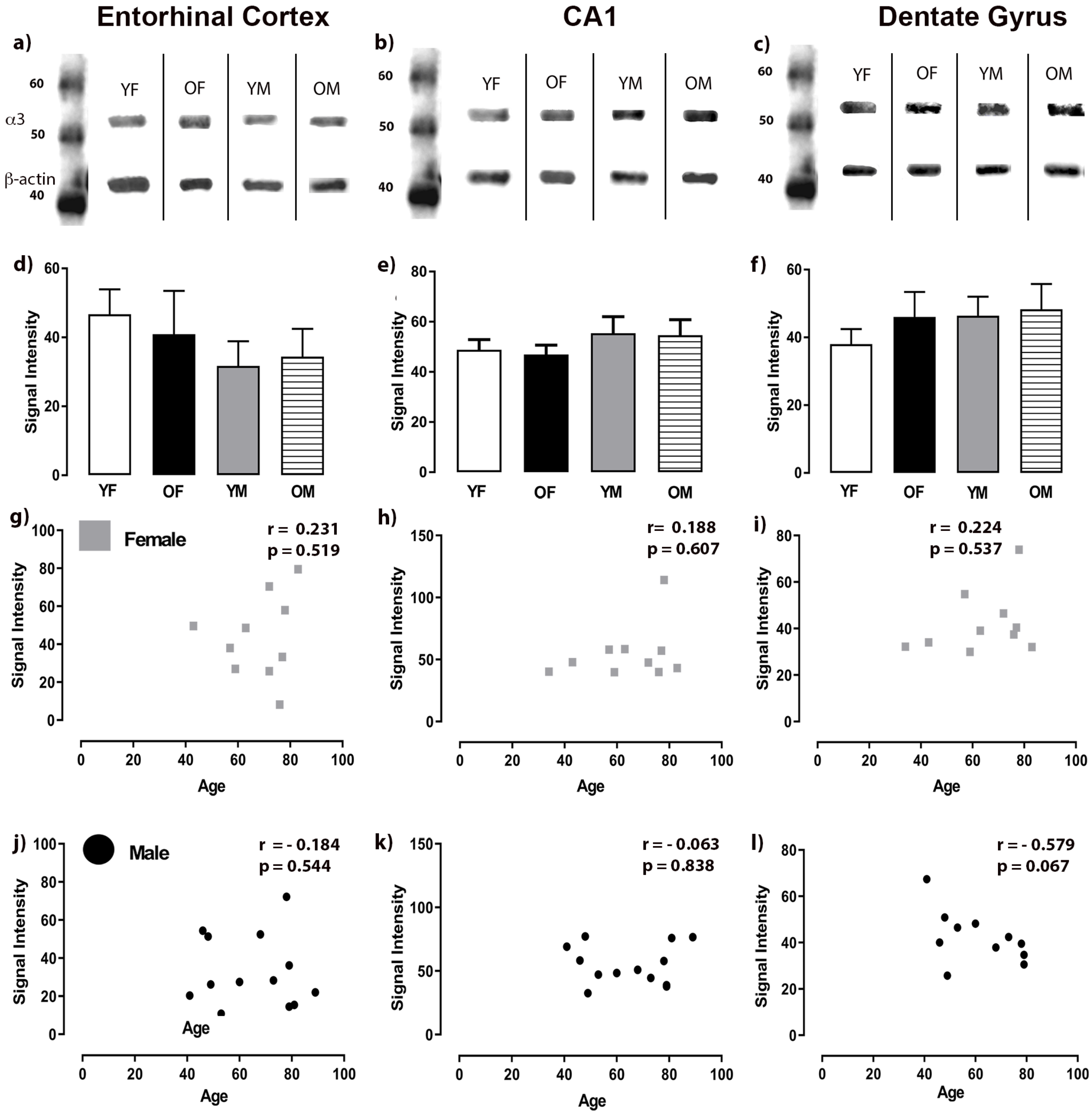

Supplementary Figure 2: **Expression of the GABA$_A$R α3 subunit in the entorhinal cortex, hippocampal CA1 region and dentate gyrus**. **a,b,c)** Representative immunoreactive Western Blot bands from younger female (YF), older female (OF), younger male (YM) and older male (OM) tissue homogenates of the ECx, CA1 and DG regions, following incubation with GABA$_A$R α3 antibody. GABA$_A$R α3 and corresponding β-actin band (α3 band size - ~55kDa, β-actin band size ~ 42 kDa) are shown. Each lane was loaded with 20µg of protein. **d,e,f)** Signal intensity graphs for each group comparing GABA α3 Western Blot band was measured and normalised to their corresponding β-actin signal for each age group. The data is graphed as mean ± SEM (N = 4-7). **g,h,i,j,k,l)** Correlation graphs for males and females plotting the relationship between age and signal intensity of GABA α3 bands.

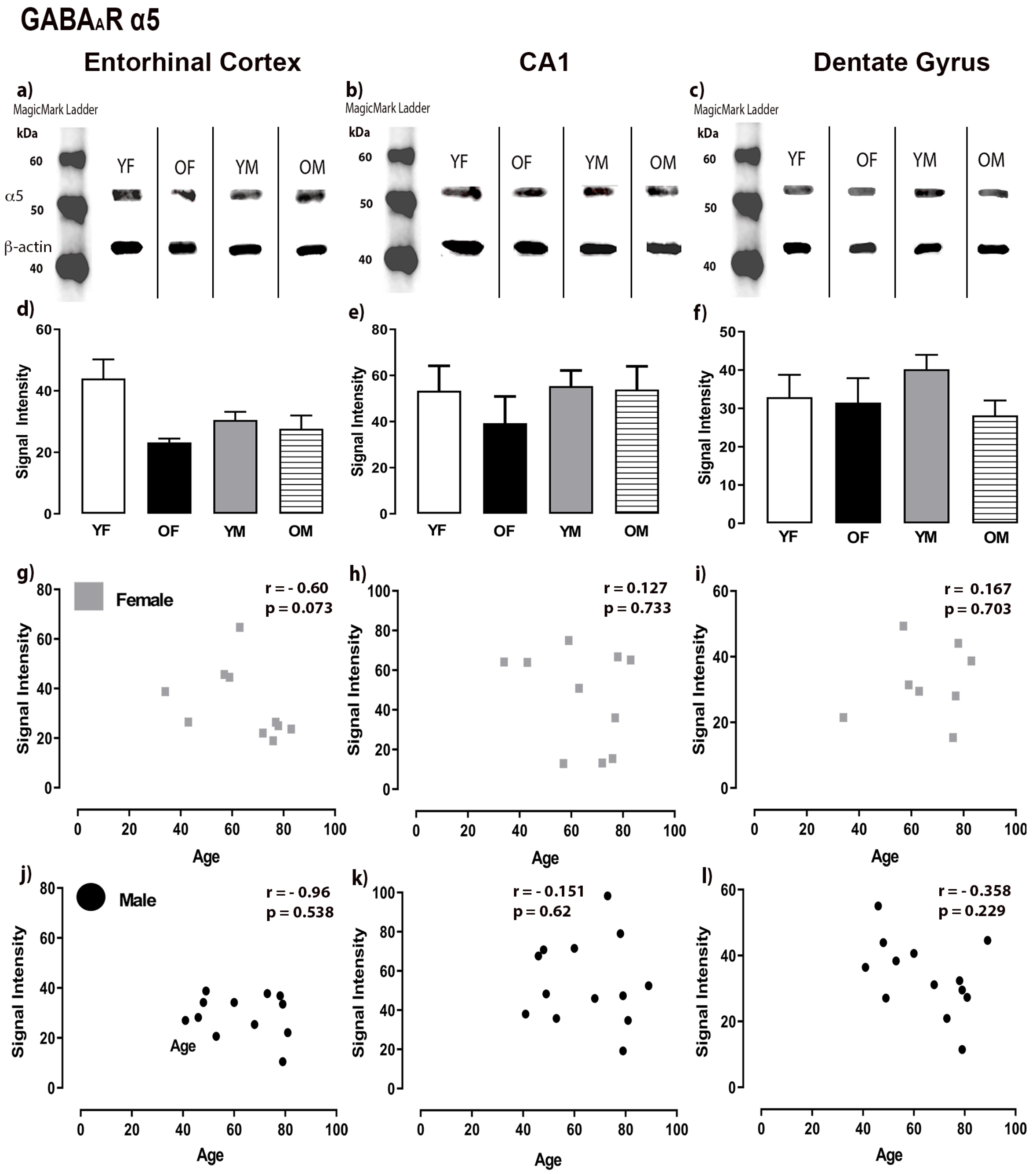

Supplementary Figure 3: Expression of the GABA$_A$R α5 subunit in the entorhinal cortex, hippocampal CA1 region and dentate gyrus. **a,b,c)** Representative immunoreactive Western Blot bands from younger female (YF), older female (OF), younger male (YM) and older male (OM) tissue homogenates of the ECx, CA1 and DG regions, following incubation with GABA$_A$R α5 antibody. GABA$_A$R α1 and corresponding β-actin band (α5 band size - ~52kDa, β-actin band size ~ 42 kDa) are shown. Each lane was loaded with 20µg of protein. **d,e,f)** Signal intensity graphs for each group comparing GABA α5 Western Blot band was measured and normalised to their corresponding β-actin signal for each age group. The data is graphed as mean ± SEM (N = 4-7). **g,h,I,j,k,l)** Correlation graphs for males and females plotting the relationship between age and signal intensity of GABA α5 bands.

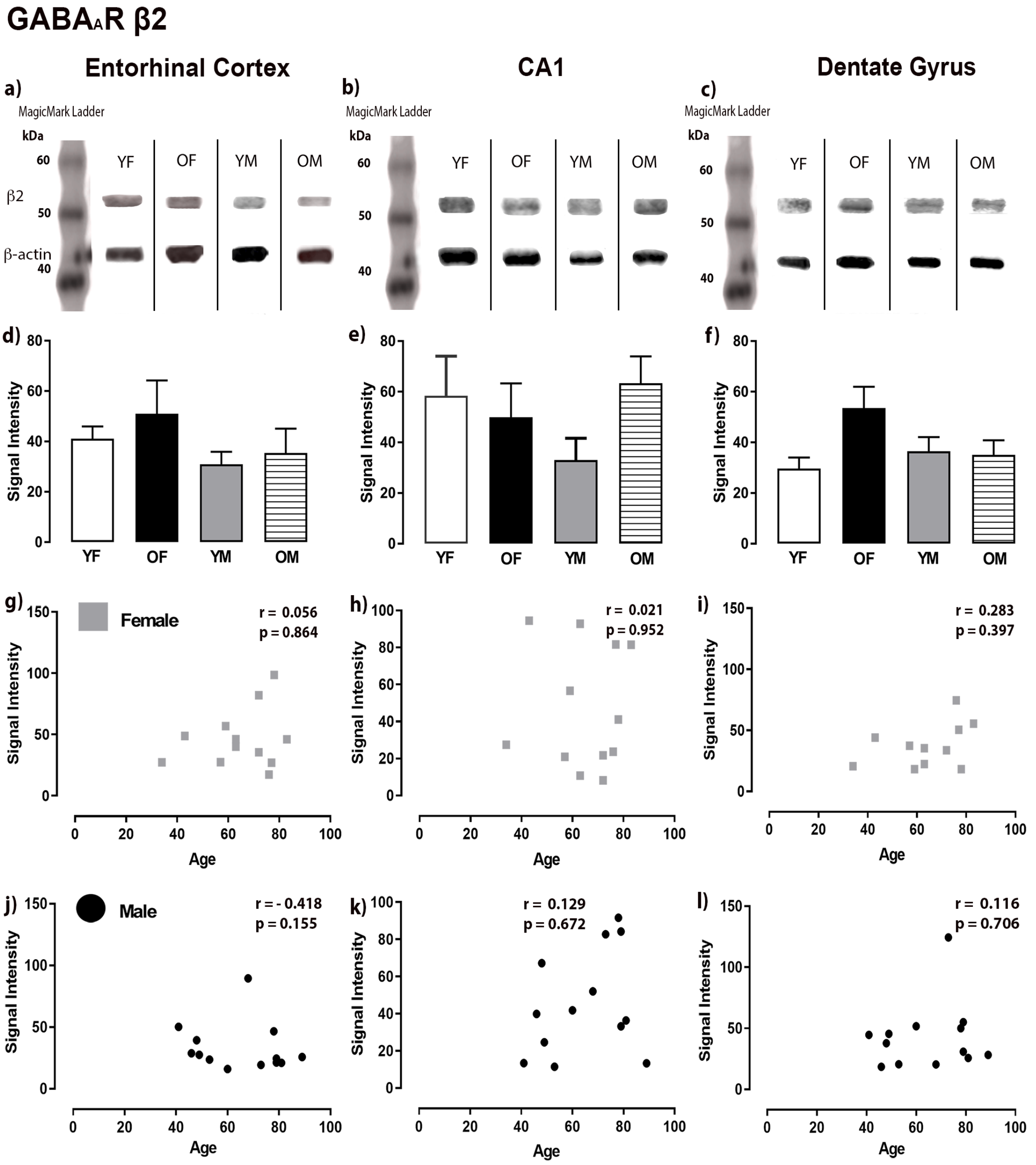

Supplementary Figure 4: Expression of the GABA$_A$R β2 subunit in the entorhinal cortex, hippocampal CA1 region and dentate gyrus. **a,b,c)** Representative immunoreactive Western Blot bands from younger female (YF), older female (OF), younger male (YM) and older male (OM) tissue homogenates of the ECx, CA1 and DG regions, following incubation with GABA$_A$R β2 antibody. GABA$_A$R β2 and corresponding β-actin band (β2 band size - ~50-53kDa, β-actin band size ~ 42 kDa) are shown. Each lane was loaded with 20μg of protein. **d,e,f)** Signal intensity graphs for each group comparing GABA β2 Western Blot band was measured and normalised to their corresponding β-actin signal for each age group. The data is graphed as mean ± SEM (N = 5-7). **g,h,i,j,k,l)** Correlation graphs for males and females plotting the relationship between age and signal intensity of GABA β2 bands.

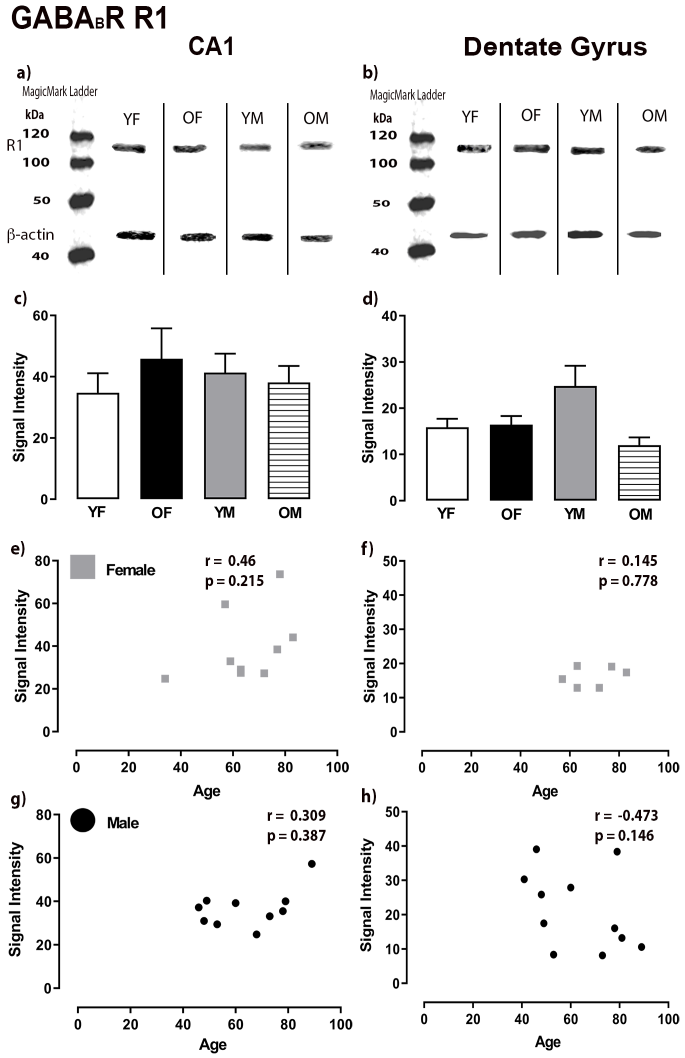

Supplementary Figure 5: Expression of the GABA$_B$R R1 subunit in the entorhinal cortex, hippocampal CA1 region and dentate gyrus. **a,b,c)** Representative immunoreactive Western Blot bands from younger female (YF), older female (OF), younger male (YM) and older male (OM) tissue homogenates of the ECx, CA1 and DG regions, following incubation with GABA$_B$R R1 antibody. GABA$_B$R R1 and corresponding β-actin band (R1 band size - ~90-120kDa, β-actin band size ~ 42 kDa) are shown. Each lane was loaded with 20µg of protein. **d,e,f)** Signal intensity graphs for each group comparing GABA$_B$R R1 Western Blot band was measured and normalised to their corresponding β-actin signal for each age group. The data is graphed as mean ± SEM (N = 4-7). **g,h,i,j,k,l)** Correlation graphs for males and females plotting the relationship between age and signal intensity of GABA$_B$R R1 bands.

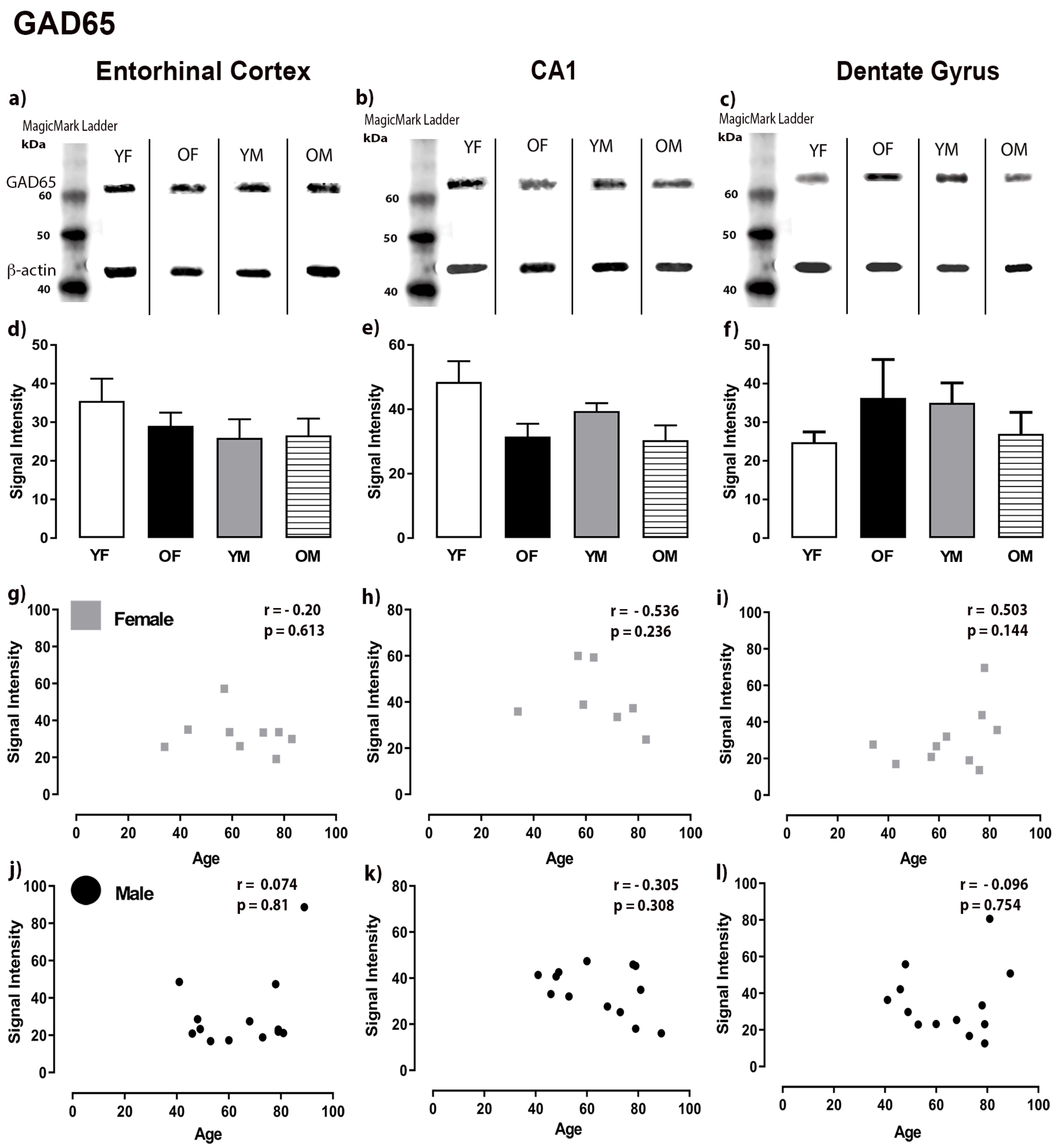

Supplementary Figure 6: Expression of the GAD 65 subunit in the entorhinal cortex, hippocampal CA1 region and dentate gyrus. **a,b,c)** Representative immunoreactive Western Blot bands from younger female (YF), older female (OF), younger male (YM) and older male (OM) tissue homogenates of the ECx, CA1 and DG regions, following incubation with GAD 65 enzyme antibody. GAD 65 and corresponding β-actin band (GAD 65 band size - ~65kDa, β-actin band size ~ 42 kDa) are shown. Each lane was loaded with 20µg of protein. **d,e,f)** Signal intensity graphs for each group comparing the GAD 65 enzyme Western Blot band was measured and normalised to their corresponding β-actin signal for each age group. The data is graphed as mean ± SEM (N = 5-7). **g,h,i,j,k,l)** Correlation graphs for males and females plotting the relationship between age and signal intensity of GAD 65 bands.

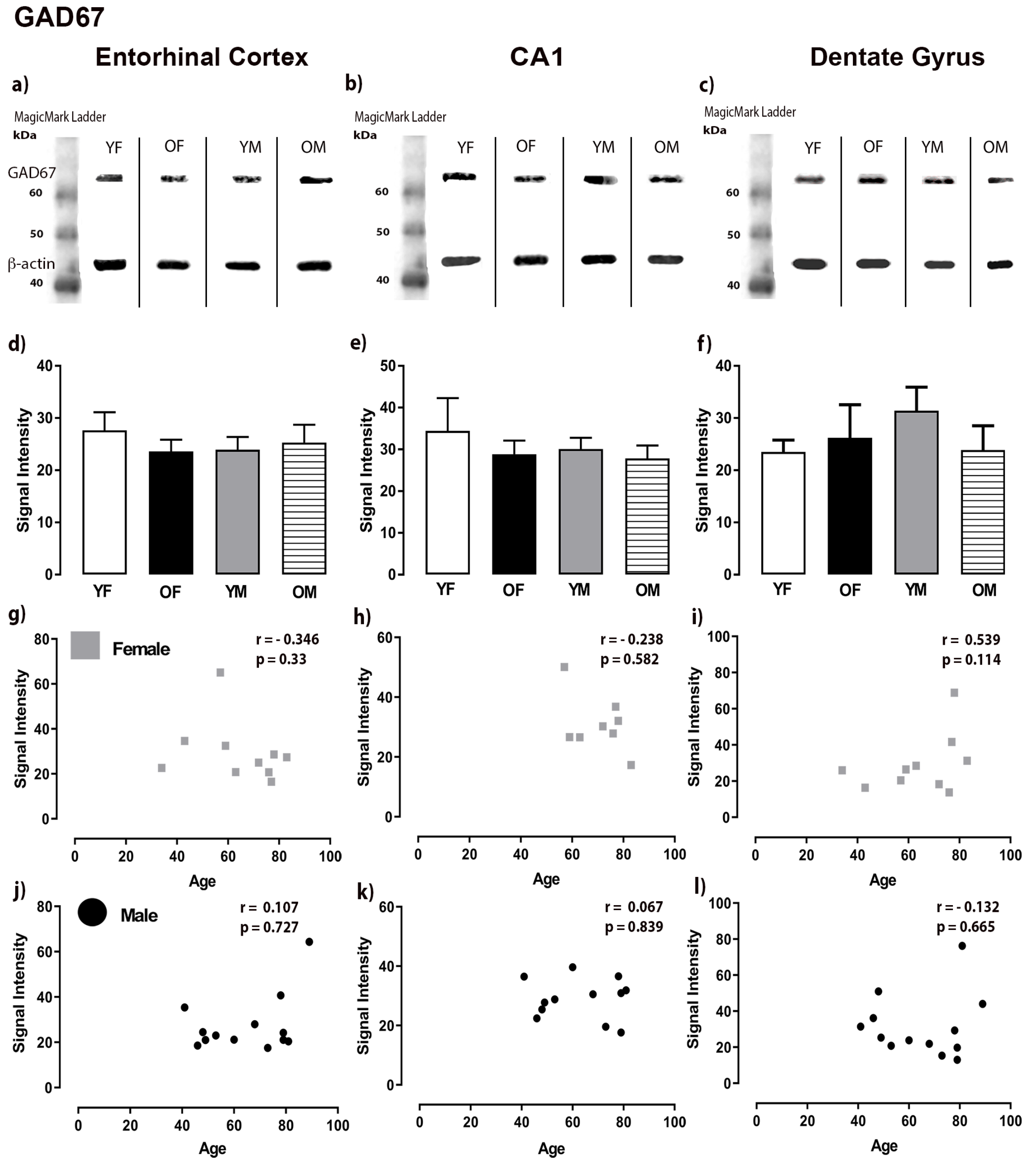

Supplementary Figure 7: Expression of the GAD 67 enzyme subunit in the entorhinal cortex, hippocampal CA1 region and dentate gyrus. **a,b,c)** Representative immunoreactive Western Blot bands from younger female (YF), older female (OF), younger male (YM) and older male (OM) tissue homogenates of the ECx, CA1 and DG regions, following incubation with GAD 67 enzyme antibody. GAD 67 and corresponding β-actin band (GAD 67 band size - ~67kDa, β-actin band size ~ 42 kDa) are shown. Each lane was loaded with 20μg of protein. **d,e,f)** Signal intensity graphs for each group comparing the GAD 67 enzyme Western Blot band was measured and normalised to their corresponding β-actin signal for each age group. The data is graphed as mean ± SEM (N = 4-7). **g,h,i,j,k,l)** Correlation graphs for males and females plotting the relationship between age and signal intensity of GAD 67 bands.

Full length blots of the representative images

GABA$_A$ α1 ETR

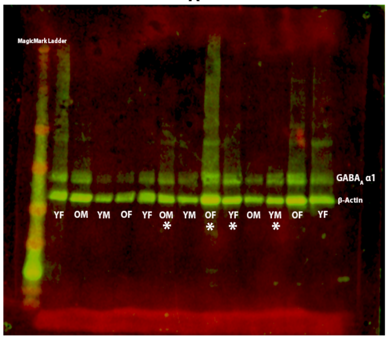

GABA$_A$ α1 CA1

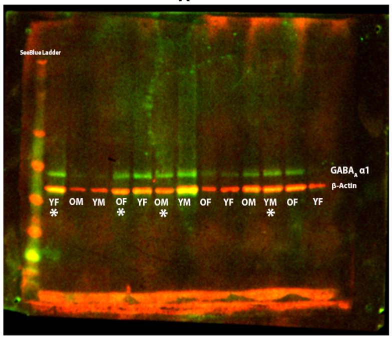

GABA$_A$ α1 DG

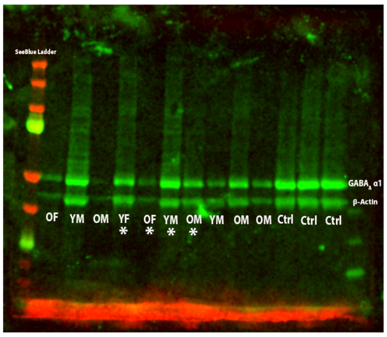

GABA$_A$ α2 ETR

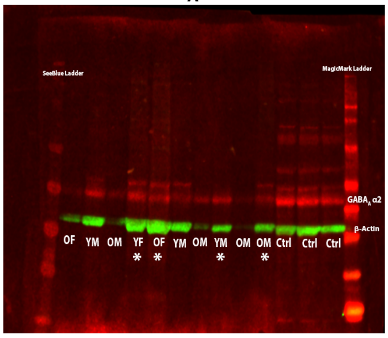

GABA$_A$ α2 CA1

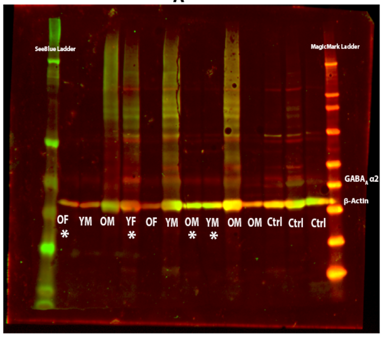

GABA$_A$ α2 DG

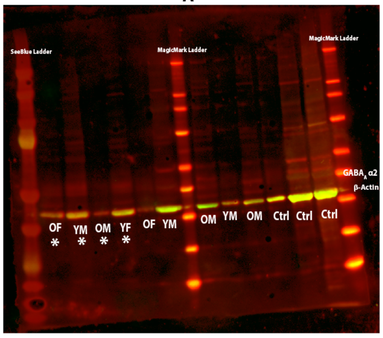

GABA$_A$ α3 ETR

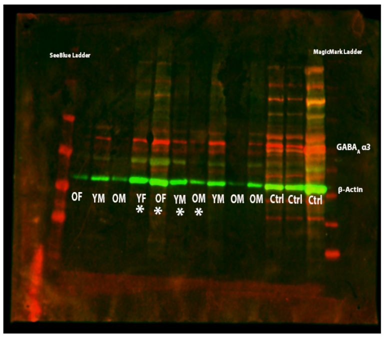

GABA$_A$ α3 CA1

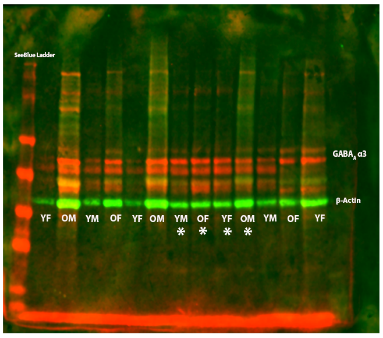

GABA$_A$ α3 DG

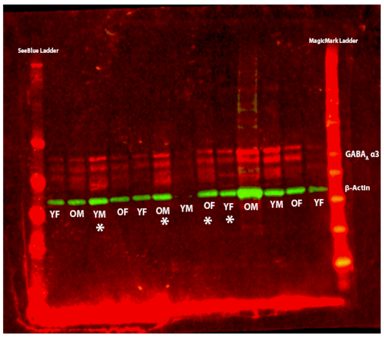

GABA$_A$ α5 ETR

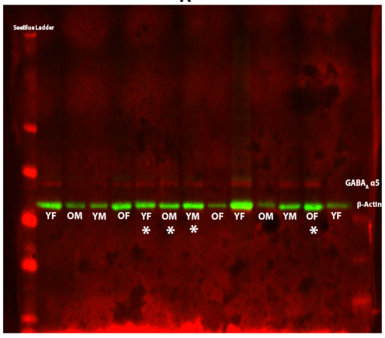

GABA$_A$ α5 CA1

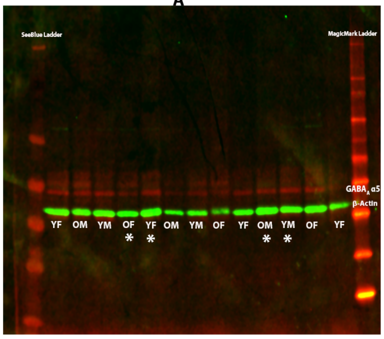

GABA$_A$ α5 DG

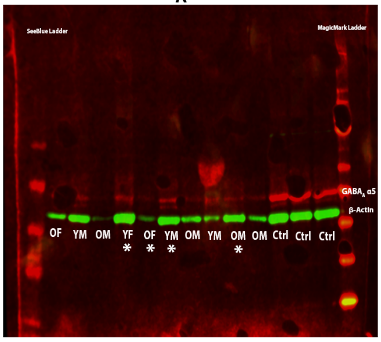

GABA$_A$ β1 ETR

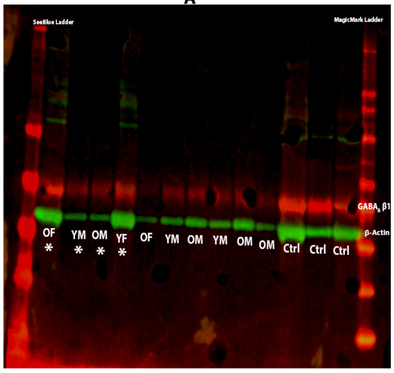

GABA$_A$ β1 CA1

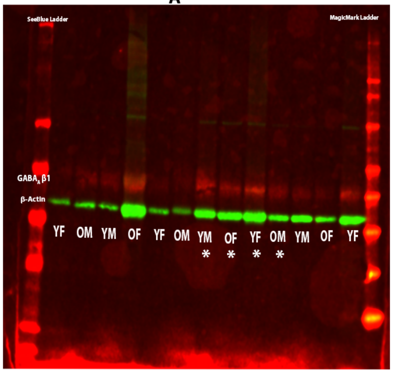

GABA$_A$ β1 DG

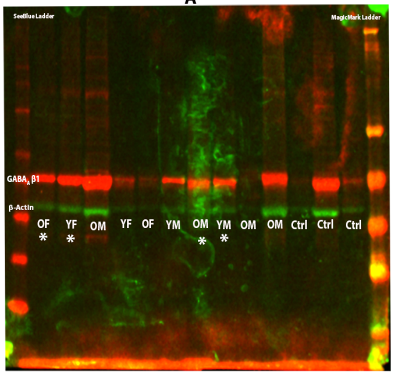

GABA$_A$ β2 ETR

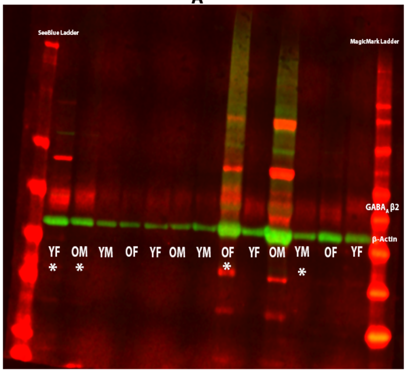

GABA$_A$ β2 CA1

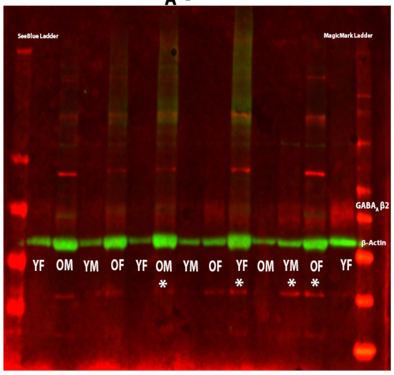

GABA$_A$ β2 DG

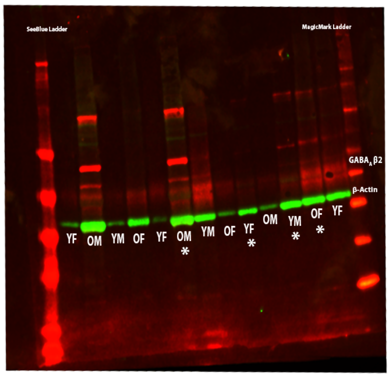

GABA$_A$ β3 ETR

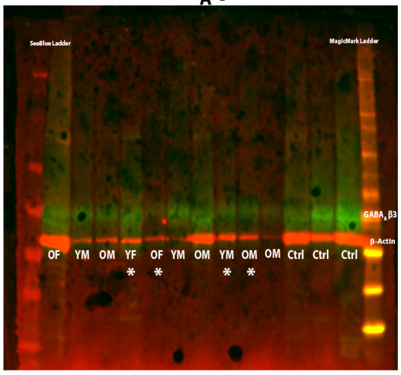

GABA$_A$ β3 CA1

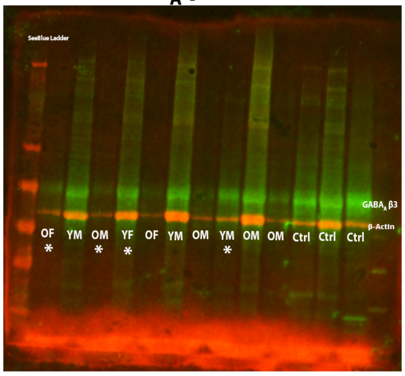

GABA$_A$ β3 DG

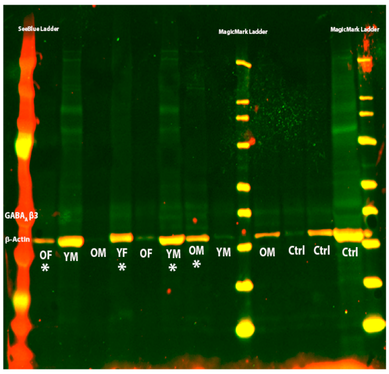

GABA$_A$ y2 ETR

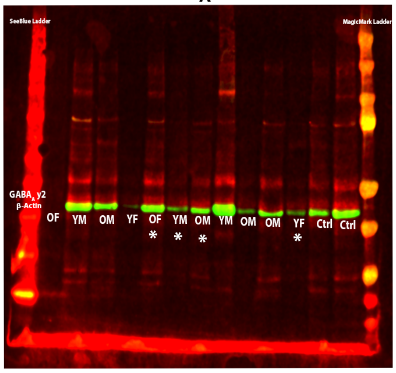

GABA$_A$ y2 CA1

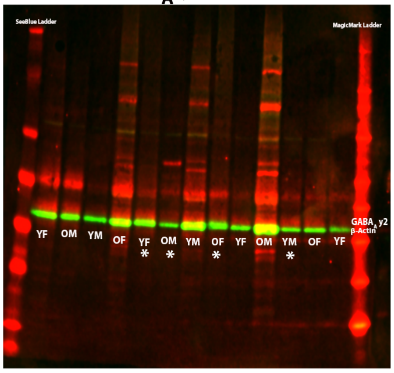

GABA$_A$ y2 DG

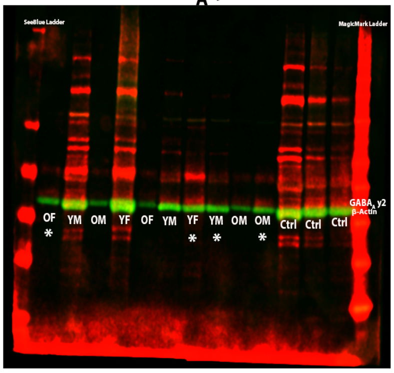

$GABA_B$ R1 ETR

$GABA_B$ R1 CA1

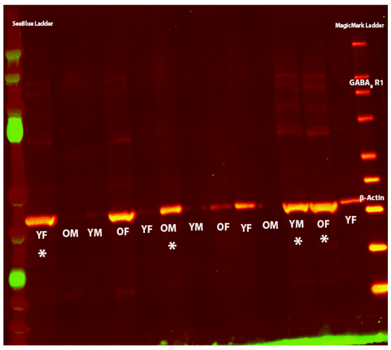

$GABA_B$ R1 DG

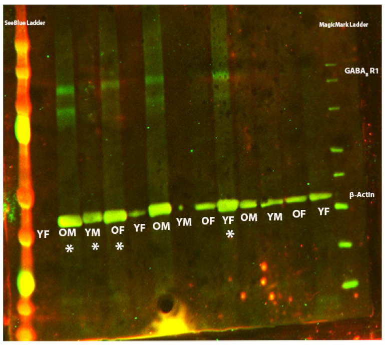

$GABA_B$ R2 ETR

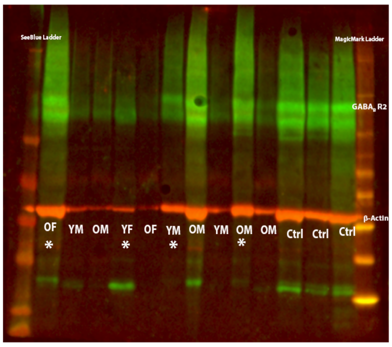

$GABA_B$ R2 CA1

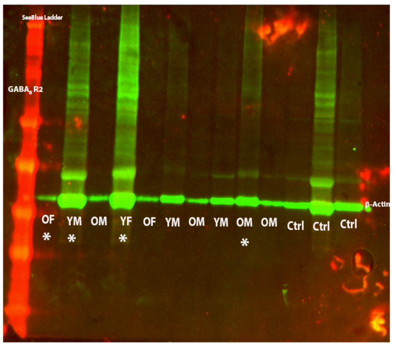

$GABA_B$ R2 DG

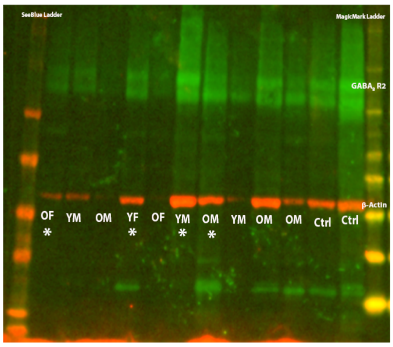

GAD 65 & 67 ETR

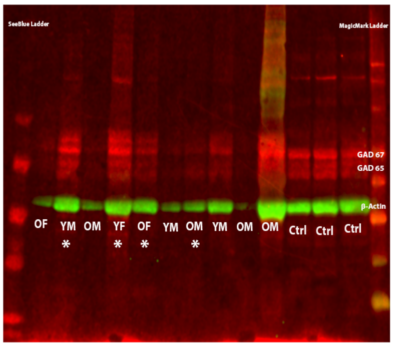

GAD 65 & 67 CA1

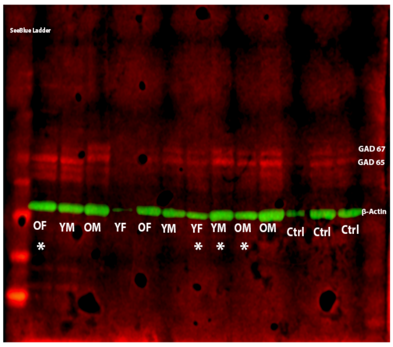

GAD 65 & 67 DG

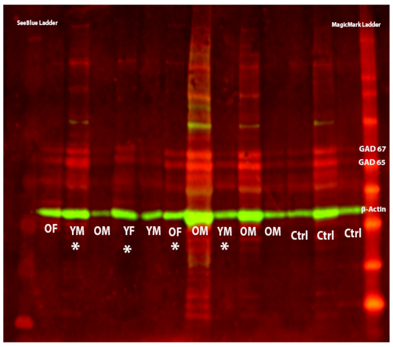

Supplement: Supplementary file 1 — Supplementary Information. [file 41598_2021_792_MOESM1_ESM.pdf]
